# Photosynthetic Entrainment of the Circadian Clock Facilitates Plant Growth under Environmental Fluctuations: Perspectives from an Integrated Model of Phase Oscillator and Phloem Transportation

**DOI:** 10.3389/fpls.2017.01859

**Published:** 2017-10-30

**Authors:** Takayuki Ohara, Akiko Satake

**Affiliations:** ^1^Graduate School of Environmental Science, Hokkaido University, Sapporo, Japan; ^2^Department of Biology, Faculty of Science, Kyushu University, Fukuoka, Japan

**Keywords:** starch degradation, sucrose, homeostasis, mathematical model, dynamics, phloem transportation, *Arabidopsis thaliana*

## Abstract

Plants need to avoid carbon starvation and resultant growth inhibition under fluctuating light environments to ensure optimal growth and reproduction. As diel patterns of carbon metabolism are influenced by the circadian clock, appropriate regulation of the clock is essential for plants to properly manage their carbon resources. For proper adjustment of the circadian phase, higher plants utilize environmental signals such as light or temperature and metabolic signals such as photosynthetic products; the importance of the latter as phase regulators has been recently elucidated. A mutant of *Arabidopsis thaliana* that is deficient in phase response to sugar has been shown, under fluctuating light conditions, to be unable to adjust starch turnover and to realize carbon homeostasis. Whereas, the effects of light entrainment on growth and survival of higher plants are well studied, the impact of phase regulation by sugar remains unknown. Here we show that endogenous sugar entrainment facilitates plant growth. We integrated two mathematical models, one describing the dynamics of carbon metabolism in *A. thaliana* source leaves and the other growth of sink tissues dependent on sucrose translocation from the source. The integrated model predicted that sugar-sensitive plants grow faster than sugar-insensitive plants under constant as well as changing photoperiod conditions. We found that sugar entrainment enables efficient carbon investment for growth by stabilizing sucrose supply to sink tissues. Our results highlight the importance of clock entrainment by both exogenous and endogenous signals for optimizing growth and increasing fitness.

## Introduction

Plants are inevitably exposed to daily and seasonal variations in light environments. To continuously grow in fluctuating environments, it is crucial for plants to stably supply carbon resources for respiration and growth. Plant growth in the day is supported by the supply of photosynthates, particularly soluble sugars such as sucrose that are transported from photosynthetic leaves (source tissues) to sink tissues (e.g., roots). Plants grow even in nighttime using carbon resources accumulated during the preceding daytime. *Arabidopsis thaliana*, a model plant, partitions a large fraction of assimilated carbon into insoluble starch, which is degraded at night to produce sucrose (Smith and Stitt, 2007; Stitt and Zeeman, 2012). Because early exhaustion of starch results in carbon starvation and ensuing growth inhibition (Graf et al., 2010; Yazdanbakhsh et al., 2011), careful management of starch metabolism is essential to cope with daily and seasonal fluctuations of light conditions.

In *A. thaliana*, starch amount increases during the day at an almost constant rate and decreases almost linearly at night (Caspar et al., 1985; Gibon et al., 2004; Smith et al., 2004; Lu et al., 2005). Plants in shorter photoperiods accumulate starch more rapidly during the day and degrade it more slowly at night than in longer photoperiods (Lu et al., 2005). Plants also adjust the rate of starch degradation immediately in response to an unexpectedly early or late onset of night (Lu et al., 2005; Graf et al., 2010; Scialdone et al., 2013). The circadian clock underlying the approximately 24-h cycle of biological processes is implicated in the control of starch metabolism (Graf and Smith, 2011). Wild type *A. thaliana* (Ws) exhausts starch reserves about 24 h after the last dawn even under non-24 h light/dark cycles (T-cycles) (Graf et al., 2010), indicating that the timing of starch exhaustion is programmed by the circadian clock. In the circadian clock mutant *cca1/lhy*, in which the functional clock has a period of about 17 h (Locke et al., 2005), the depletion of starch occurs prematurely under a 24-h T-cycle but coincides with dawn under a 17-h T-cycle (Graf et al., 2010). These studies suggest that coordination of the internal timing of starch turnover with environmental cycles is necessary to avoid carbon starvation.

Phase adjustment of the circadian clock to external stimuli such as light or temperature is fundamental for synchronizing biological processes with environments (Johnson et al., 2003). In addition to signals from the external environment, endogenous signals such as photosynthates are also important regulators of the circadian phase in *A. thaliana*. We previously reported that phase adjustment by sugar is necessary for plants to flexibly regulate carbon metabolism in fluctuating light environments (Seki et al., 2017). We developed a phase oscillator model describing phase regulation of the circadian clock by sucrose. This model predicted that phase adjustment of the circadian clock by sucrose is crucial for homeostatic regulation of carbon resources. These theoretical predictions were confirmed by physiological experiments using the mutant *pseudoresponse regulator 7–11* (*prr7–11*), the circadian clock of which does not show clear phase response to sucrose pulse (Haydon et al., 2013).

Whereas, clock entrainment by exogenous signals such as light has been shown to be advantageous for competition and survival in several organisms (Woelfle et al., 2004; Dodd et al., 2005), the advantages of clock entrainment by photosynthetic products remain elusive. Here we theoretically evaluate the effects of clock entrainment by sugar on plant growth, a good proxy for plant fitness (Younginger et al., 2017). We extended the phase oscillator model for the circadian clock (Seki et al., 2017) by incorporating growth dynamics of sink tissues, including shoot apical meristems and roots. Growth dynamics are described by modeling phloem transportation of sucrose from source to sink tissues (Seki et al., 2015; Satake et al., 2016). We demonstrate that plant growth is facilitated by endogenous sugar entrainment under long photoperiods because the entrainment enables the stable supply of sucrose from source to sink tissues irrespective of light fluctuation. In short photoperiods, however, the effect of sugar entrainment on growth is negligibly small. Our results provide important theoretical evidence that circadian-phase adjustment by endogenous signals is advantageous for plant growth.

## Model and methods

**Figure 1:**
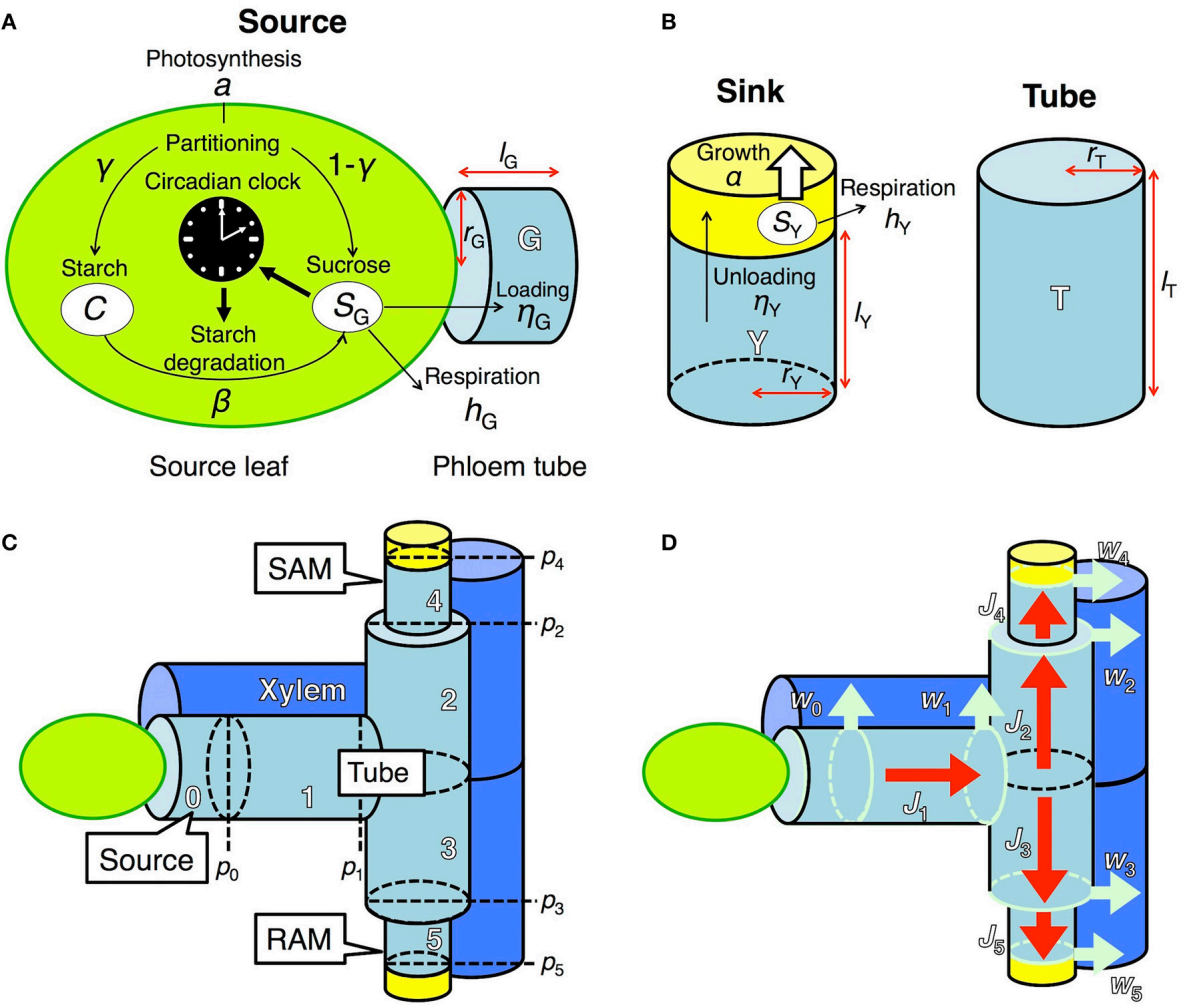
Model describing sugar dynamics in source and sink tissues and translocation of sucrose through the phloem tube. **(A)** In a source leaf, the circadian clock and carbon metabolism are reciprocally regulated. **(B)** Translocated sucrose is used for growth and respiration in the sink. Each structural component is expressed by a cylinder of radius *r*_X_ and height *l*_X_, where X is either G (source), T (tube), or Y (sink). **(C)** Tubal structure of the model. *p*_*i*_ represents hydrostatic pressure at the apex of component *i* (*i* ϵ {0,…, 5}). Xylem adjacent to the phloem tube is also schematized. SAM, shoot apical meristem; RAM, root apical meristem. **(D)** Fluxes in the model. Red arrows (*J*_*i*_) indicate phloem sap flow. Light blue arrows (*w*_*i*_) indicate pure water flow due to osmosis, which occurs at the region represented by light blue circles.

To investigate the effect of clock regulation by sugar on plant growth, we integrated two previously developed models, one describing the dynamics of starch and sucrose metabolism in source leaves (Seki et al., 2017) and the other growth of sink tissues dependent on phloem transportation of sucrose from source tissues (Seki et al., 2015; Satake et al., 2016). Photosynthetic products in the source leaf are partitioned into sucrose and starch (Seki et al., 2017; Figure 1A). Sucrose in the source leaf is loaded into the phloem, moves through the phloem tube, and is unloaded at sink tissues where it is used for respiration and growth (Seki et al., 2015; Satake et al., 2016; Figures 1B–D). These two models were coupled by incorporating a term that represents sucrose translocation from source to sink. We explain the detailed structures of each component of our new integrated model in the following sections. We consider two sink tissues, the shoot and root apical meristems (SAM and RAM, respectively), because plant growth and development mainly occur in these organs (Figure 1C). Our model can be extended to the structure including multiple sinks in the complex phloem network as studied previously (Seki et al., 2015; Satake et al., 2016).

### Sugar dynamics in source leaves and sink tissues

#### Source leaves

In source leaves (Figure 1A), carbon is assimilated by photosynthesis at a rate *a* during the light period. The length of the light period is given by τ_L_. A fraction γ of total photoassimilates is partitioned into starch (*C*) for storage and a fraction 1 − γ is partitioned into sucrose (*S*_G_). Sucrose is consumed for respiration at a rate *h*_G_ and for transportation at a rate η_*G*_. Starch is degraded into sucrose at a rate β, which is assumed to be under the control of the circadian clock and thus is a function of the phase ϕ of the circadian oscillator (Seki et al., 2017). These processes are formalized as follows:

(1) $$\begin{array}{l} \frac{d}{dt}S_{G}\left(t\right)=aL\left(t\right)\left(1-\gamma \right)+\beta \left(\phi \right)C^{\kappa }-\left(h_{G}+\eta_{G}\right)S_{G}, \end{array}$$

(2) $$\begin{array}{l} \frac{d}{dt}C\left(t\right)=aL\left(t\right)\gamma -\beta \left(\phi \right)C^{\kappa }, \end{array}$$

where *L*(*t*) indicates the light condition (defined as 1 under light and 0 under dark) and κ is a constant. Starch reserve is accumulated during the light period at a rate determined by balance between *a*γ and β*C*^κ^. The starch degradation rate β(ϕ) is assumed to show diel oscillation due to regulation by the circadian clock. We assume that β(ϕ) shows a peak at dawn and a trough at the subjective dusk ϕ^*^ because a previous study showed that this oscillation pattern is ideal to minimize fluctuations in the sucrose supply to sinks (Figure S1A; Seki et al., 2017).

In the previous model (Seki et al., 2017), transportation of sucrose from source leaves was assumed to be constant. In our new model, sucrose transportation is assumed to occur based on the pressure-flow hypothesis (Münch, 1930) with an assumption that the flux in phloem obeys the Hagen–Poiseuille law (Supplementary Material, section 1). Sucrose dynamics are now described both in the source leaves and phloem tubes. At the source leaves, sucrose is loaded into the adjacent phloem tube at a rate η_G_ (Figure 1A). At the phloem tube, loaded sucrose is transported to sink tissues (see Supplementary Material, section 1 for detailed explanation). The dynamics of sucrose concentration at the phloem tube adjacent to the source (*g*_0_) is given by

(3) $$\frac{d}{dt}g_{0}\left(t\right)=\frac{1}{V_{0}}\{\eta_{G}S_{G}\left(t\right)+g_{1}\left(t\right){\left[-J_{1}\left(t\right)\right]}_{+}-g_{0}\left(t\right){\left[J_{1}\left(t\right)\right]}_{+}\},$$

where *g*_1_(*t*)[–*J*_1_(*t*)]_+_ − *g*_0_(*t*)[*J*_1_(*t*)]_+_ describes the rate of sucrose change due to flux *J*_1_(*t*) at phloem tube 1 (Figures 1C,D). *g*_1_ and *V*_0_ represent sucrose concentration at tube 1 and the volume of tube 0, respectively (Figure 1C).

Similar to the previous model (Seki et al., 2017), the phase ϕ of the circadian oscillator is modeled by

(4) $$\begin{array}{l} \frac{d}{dt}\phi \left(t\right)=\omega +Z_{L}\left(\phi \right)f_{L}\left(\tilde{L}\right)+Z_{S}\left(\phi \right)f_{S}\left({\tilde{S}}_{G}\right), \end{array}$$

where ω is the angular frequency of the oscillator. The term *Z*_L_(ϕ)*f*_L_($\tilde{L}$) represents the effect of light stimulus, which is assumed to reset the phase to 0 at dawn and to τ_L_ at dusk (Seki et al., 2017). *Z*_S_(ϕ) is a phase response curve (PRC) to a sucrose pulse showing phase advance until ϕ = ϕ^*^ and phase delay thereafter as previously determined (Figure S1B; Seki et al., 2017). Sugar input *f*_S_ (Supplementary Material, section 2) is defined by the Hill function of the rate of change in sucrose level (i.e., ${\tilde{S}}_{G}=dS_{G}/dt$). The functions of *Z*_S_(ϕ) and *f*_S_ have been demonstrated to be optimal for minimizing sucrose fluctuation (Seki et al., 2017). We assume that the phase shift of the circadian clock by sugar takes place only in the light period because the phase shift at night did not improve sucrose homeostasis (Seki et al., 2017).

#### Sink tissues

Translocated sucrose is unloaded into the sinks at a rate η_Y_ from the adjacent phloem tubes with sucrose concentration *g*_*i*_ (Figure 1B). Sucrose in the sink (*S*_Y_) is consumed for respiration and growth at rates *h*_Y_ and α(*S*_Y_), respectively. These processes are formalized as follows:

(5) $$\begin{array}{l} \frac{d}{dt}g_{i}\left(t\right)=\frac{1}{V_{i}}\left\{g_{i-2}\left(t\right){\left[J_{i}\left(t\right)\right]}_{+}-g_{i}\left(t\right)\left({\left[-J_{i}\left(t\right)\right]}_{+}+\eta_{\text{Y}}\right)\right\}, \end{array}$$

(6) $$\begin{array}{l} \frac{d}{dt}S_{\text{Y}}\left(t\right)=\eta_{\text{Y}}g_{i}\left(t\right)-h_{\text{Y}}S_{\text{Y}}\left(t\right)-\alpha \left(S_{\text{Y}}\right), \end{array}$$

where *V*_*i*_ is the volume of component *i* (*i* = 4 for SAM and *i* = 5 for RAM; *i* = 1, 2, and 3 for connecting tubes between the source and sink; Figure 1C). The first and second terms in the right-hand side of Equation (5) represent the solution inflow and outflow, respectively (see Supplementary Material, section 1). The function for sucrose consumption rate for growth α(*S*_Y_) will be explained in the next subsection. To clarify the effect of sugar entrainment on plant growth, we simplified the model by assuming that the sugar and growth dynamics of SAM and RAM are identical. Therefore, the values of η_Y_ and *h*_Y_ as well as the parameters in α(*S*_Y_) are the same in the two sinks.

### Growth of sink tissues

#### Growth dynamics

**Figure 2:**
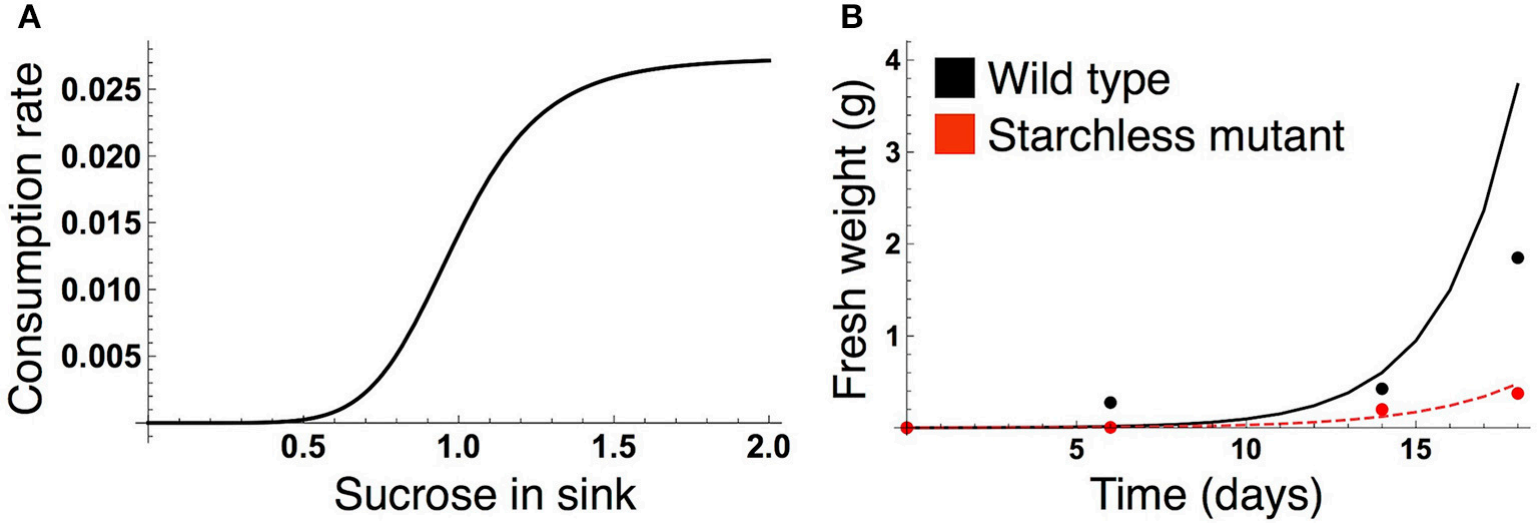
**(A)** Sucrose consumption rate α for growth of the sink (SAM or RAM). The unit for sucrose is μmolC_6_ g^−1^FW. **(B)** Time evolution of growth of the wild type (black) and the starchless *pgm* mutant (red) grown in a 12-h photoperiod. In **(B)**, lines represent the sum of the simulated growth of the two sinks and circles represent the published growth data (Caspar et al., 1985).

We formalized the growth kinetics of the sink tissue based on sucrose supply because a strong correlation between growth rate and sucrose supply has been reported in both the light and dark periods for *A. thaliana* (Sulpice et al., 2014; Mengin et al., 2017). When sugar supply is sufficient, growth is promoted by the target of rapamycin (TOR) kinase, the expression level of which correlates with *A. thaliana* shoot and root growth (Deprost et al., 2007; Lastdrager et al., 2014). On the contrary, Snf1-related kinase 1 (SnRK1) inhibits growth in response to low carbon availability (Baena-González and Sheen, 2008; Lastdrager et al., 2014). In addition, growth rate is likely to be saturated as sucrose supply is increased (Sulpice et al., 2014). Given these empirical findings, we assume that growth-related sucrose consumption rate is an increasing and saturating function of sucrose supply (Figure 2A):

(7) $$\begin{array}{l} \alpha \left(S_{\text{Y}}\right)=\alpha_{\text{Max}}\frac{S_{\text{Y}}^{ñ}}{{\tilde{K}}^{ñ}+S_{\text{Y}}^{ñ}}, \end{array}$$

where α_Max_, ñ, and $\tilde{K}$ are constants. These values are estimated using the published data of fresh biomass in *A. thaliana* (Caspar et al., 1985) as explained later. The rate of increase in sink fresh biomass (*W*_Y_) is then described by

(8) $$\begin{array}{l} \frac{d}{dt}W_{\text{Y}}\left(t\right)=\lambda \alpha \left(S_{\text{Y}}\right)W_{\text{Y}}\left(t\right), \end{array}$$

where λ is the conversion rate of sucrose for growth. Therefore, growth rate is represented by λα(*S*_Y_). Since dry weight and fresh weight of *A. thaliana* Col-0 display qualitatively similar increase patterns (Caspar et al., 1985; Christophe et al., 2008), our model can also be applicable to the analysis of dry biomass by appropriate scaling. We tested the robustness of our results by using an alternative formalization of growth rate as a linear increasing function of sucrose (Supplementary Material, section 3; Figures S4, S5).

#### Parameter estimation of the sucrose consumption rate for growth

The parameter values of the sucrose consumption rate α(*S*_Y_) in Equation (7) were estimated by fitting the simulated growth curves to published data measuring fresh weight of *A. thaliana* wild type (Col-0) and the starchless *phosphoglucomutase* (*pgm*) mutant grown in a 12-h photoperiod (Caspar et al., 1985; Figure 2B). Because the number of the data is limited (data at four time points per genotype), we combine the data of both genotypes for the parameter estimation. The subjective dusk ϕ^*^ was set to 12 h because both the wild type and *pgm* were likely to be completely entrained to a 12-h photoperiod in the experiment. We simulated the growth of the wild type (see next subsection) and starchless mutant using an initial value of 0.0005 at time *t* = 0 (corresponding to the first observation of plant fresh weight in the experiment). Because the *pgm* mutant does not accumulate starch, the carbon partitioning rate for starch (γ in Equations 1 and 2) was set to 0 for the mutant. The parameter values α_Max_, ñ, and $\tilde{K}$ in Equation (7) were estimated by minimizing a following cost function:

(9) $$\begin{array}{l} P(\alpha_{\text{Max}},ñ,\tilde{K})=\sum_{X}\sum_{i=1}^{4}{\left\{W_{i}^{EXP}-2W_{Y,i}\left(\alpha_{\text{Max}},ñ,\tilde{K}\right)\right\}}^{2}, \end{array}$$

where $W_{i}^{EXP}$ and *W*_Y,*i*_ are fresh weight at the *i*th time point in the experiment and the growth at corresponding times in the simulation, respectively (Figure 2B). *X* corresponds to the wild type and *pgm* mutant. As fresh weight is likely to be measured from whole plants in the experiment (Caspar et al., 1985), we used the sum (2*W*_Y,*i*_) of the growth of SAM (*W*_Y,*i*_) and RAM (also *W*_Y,*i*_) for the parameter estimation.

#### Simulation conditions

To evaluate the effect of clock entrainment by sugar on growth, we simulated the growth dynamics of sugar-sensitive (wild type) and sugar-insensitive (mutant) plants in constant photoperiods (ranging from 8 to 16 h) as well as under changing photoperiod conditions. The wild type adjusts the phase of the circadian clock by sugar as formalized in Equation (4), while the sugar-insensitive mutant lacks this response to sugar [i.e., *f*_S_(${\tilde{S}}_{\text{G}}$) in Equation (4) is zero]. Both plants respond to light signals in the same manner. Although phase regulation by sugar reduces fluctuation of carbon resources, sucrose dynamics still deviate from homeostasis in both the wild type and mutant unless these plants precisely predict the timing of dusk (i.e., ϕ^*^ = τ_L_; Seki et al., 2017). To investigate the effect of sucrose homeostasis on growth, we also consider an ideal plant that can maintain perfect homeostasis in a steady state in any photoperiod (hereafter termed the homeostatic plant). We previously determined the optimal function for starch degradation rate to minimize sucrose fluctuation in a given photoperiod (Seki et al., 2017). The homeostatic plant is assumed to possess these functions in each photoperiod and is simulated by setting ϕ^*^ being equal to τ_L_ in any condition (τ_L_ ϵ(0, 24]) in contrast to the fixed value of ϕ^*^ in the wild type and sugar-insensitive mutant (summarized in a table in Figure S1). Comparison of growth dynamics among these three plants enables us to address the differential effects of endogenous sugar entrainment and sucrose homeostasis on growth. Because SAM and RAM grow at similar rates, we present the growth dynamics of SAM as the sink growth.

Under the constant photoperiod conditions, plant growth was simulated over 10 days at various photoperiods (8, 9, 10, …, 16 h). We then compared the growth increment over 10 days among plant types by calculating the differences for the following pairs: (wild type—mutant), (homeostatic—mutant), and (homeostatic—wild type). Under the changing photoperiod conditions, plants grown for 5 days in an 8-h or 16-h photoperiod were transferred to a 16-h or 8-h photoperiod and grown for an additional 5 days (Seki et al., 2017). For the wild type and sugar-insensitive mutant, we mainly analyzed the plants with the subjective dusk ϕ^*^ of 10 h. In addition, we also simulated 10-day growth of the plants with various values of ϕ^*^ (8, 9, 11, 12 h) in constant photoperiods to investigate the influence of ϕ^*^ on growth dynamics. Other parameter values are listed in Table S1. Numerical integration of the ordinary differential equations was performed with the fourth-order Runge-Kutta method using Mathematica (version 10; Wolfram Research).

## Results

**Figure 3:**
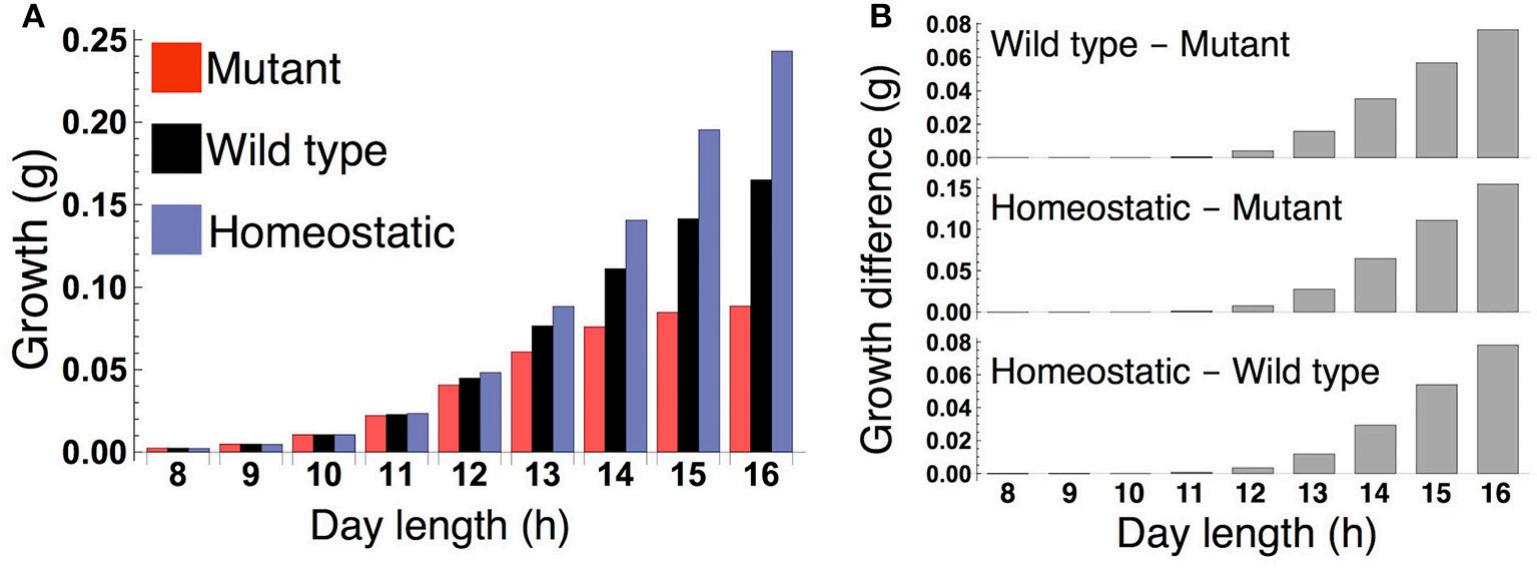
**(A)** Growth of the sink (SAM or RAM) of the mutant, wild type, and homeostatic plant over 10 days under constant photoperiod conditions. **(B)** Growth difference between the wild type and mutant (upper), between the homeostatic and mutant (middle), and between the homeostatic and wild type (lower).

When the plants are grown in a constant photoperiod, the growth of the sink (SAM or RAM) of the all three types increases as the photoperiod is lengthened (Figure 3A). The sugar-sensitive wild type and homeostatic plant grow significantly faster than the sugar-insensitive mutant under long days (Figure 3B). When photoperiod is shorter than 12 h, the growth difference between the mutant and the others almost disappears. Growth of the wild type is slower than that of the homeostatic plant in long photoperiods (Figure 3B), suggesting that the minimization of sucrose fluctuation is the most effective strategy for efficient growth under these conditions. We confirmed the robustness of our results under different parameter values for carbon metabolism (Figure S2) as well as for the phloem tube network (Figure S3).

**Figure 4:**
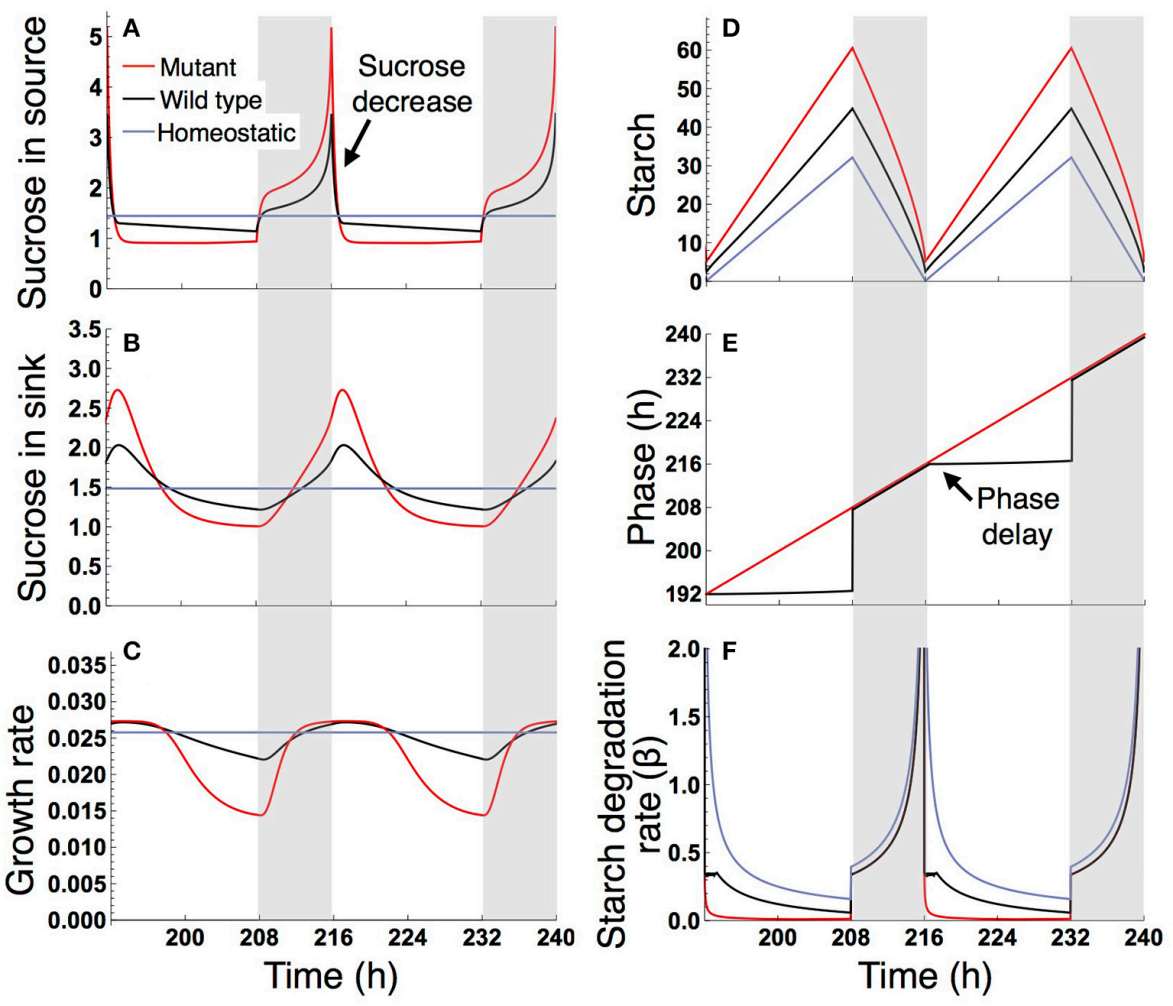
Predicted profiles of **(A)** sucrose in the source, **(B)** sucrose in the sink (SAM or RAM), **(C)** growth rate of the sink (SAM or RAM), **(D)** starch, **(E)** phase, and **(F)** starch degradation rate of the plants grown in a 16-h photoperiod. The unit for sucrose and starch is μmolC_6_ g^−1^FW. White background, light period; Gray background, dark period.

Under a long day (16 L/ 8 D), sucrose levels in the source and sink (SAM or RAM) are highly variable in the sugar-insensitive mutant, moderately variable in the wild type, and almost constant in the homeostatic plant (Figures 4A,B). Diel patterns of growth reflect sucrose profiles in the sink, revealing a substantially lower growth rate during the evening in the mutant and a moderately lower growth rate during the evening in the wild type compared to the homeostatic plant (Figure 4C). The difference in evening growth rate is the major reason for differential growth among the three plant types in a long photoperiod. The mutant accumulates the largest amount of starch, with some amount unused even at the end of night (Figure 4D), indicating inefficient translocation of carbon. In contrast, the wild type accumulates less starch than the mutant and invests a larger amount of photoassimilate for growth (Figure 4D).

The different sucrose, starch, and growth profiles between the wild type and sugar-insensitive mutant are caused by the clock plasticity in response to sugar. The decrease in sucrose levels at dawn (Figure 4A) is sensed by the wild type as a negative sugar signal, driving a phase delay of the circadian oscillator (Figure 4E). This phase delay in the morning increases the starch degradation rate during the light period (Figure 4F), resulting in elevation of the sucrose level (Figure 4A) and decrease of the starch level (Figure 4D). Such a phase shift in the circadian oscillator does not occur in the mutant (Figure 4E). Excessively high sucrose in the mutant around dawn (Figure 4B) does not significantly contribute to growth (Figure 4C) due to the saturating property of the growth-related sucrose consumption rate α (Figure 2A), while the sucrose decrease around dusk substantially reduces growth rate since at this low level α is almost linearly dependent on sucrose.

**Figure 5:**
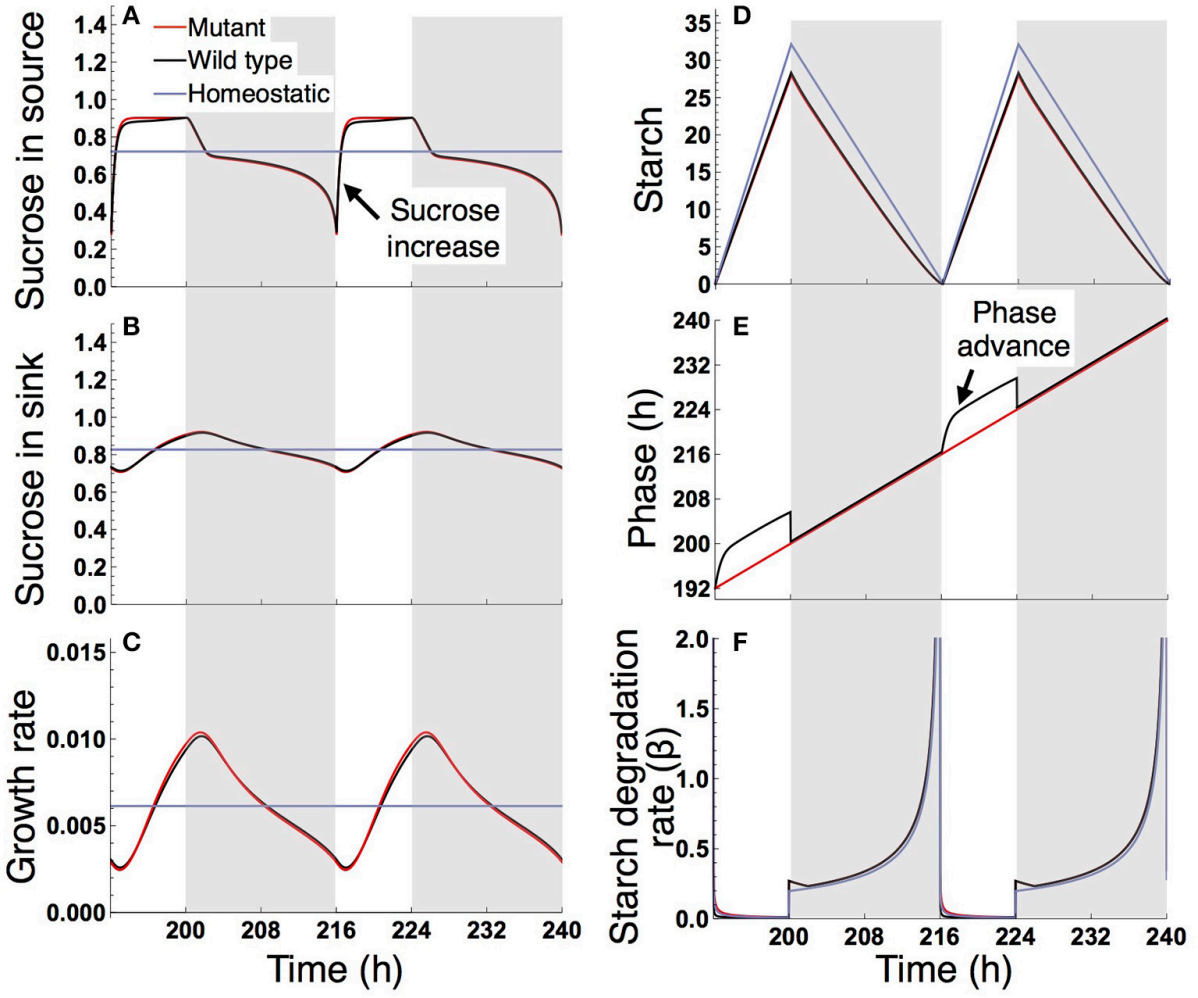
Predicted profiles of **(A)** sucrose in the source, **(B)** sucrose in the sink (SAM or RAM), **(C)** growth rate of the sink (SAM or RAM), **(D)** starch, **(E)** phase, and **(F)** starch degradation rate of the plants grown in an 8-h photoperiod. Details are as in the legend to Figure 4.

Under a short day (8 L/16 D; Figure 5), the wild type and sugar-insensitive mutant show the similar sugar and growth dynamics as reported previously (Seki et al., 2017). The elevation of sucrose at dawn (Figure 5A) gives rise to a positive sugar signal and resultant phase advance in the wild type (Figure 5E). However, this phase shift does not substantially change the starch degradation rate (Figure 5F), so the sugar and growth dynamics are almost the same for the wild type and mutant (Figures 5A–D). The homeostatic plant displays distinct growth dynamics (Figure 5C). Nevertheless, the difference in 10-day growth among plants is very small in the short photoperiod (Figure 3B). Since all three plants fix less carbon per day compared to under long day conditions, sucrose concentration is low both in the source and sink (Figures 5A,B). This leads to a slow growth rate due to low supply of sucrose. Because all three plants are limited by sucrose under short day conditions, the growth difference among them is indistinguishably small.

**Figure 6:**
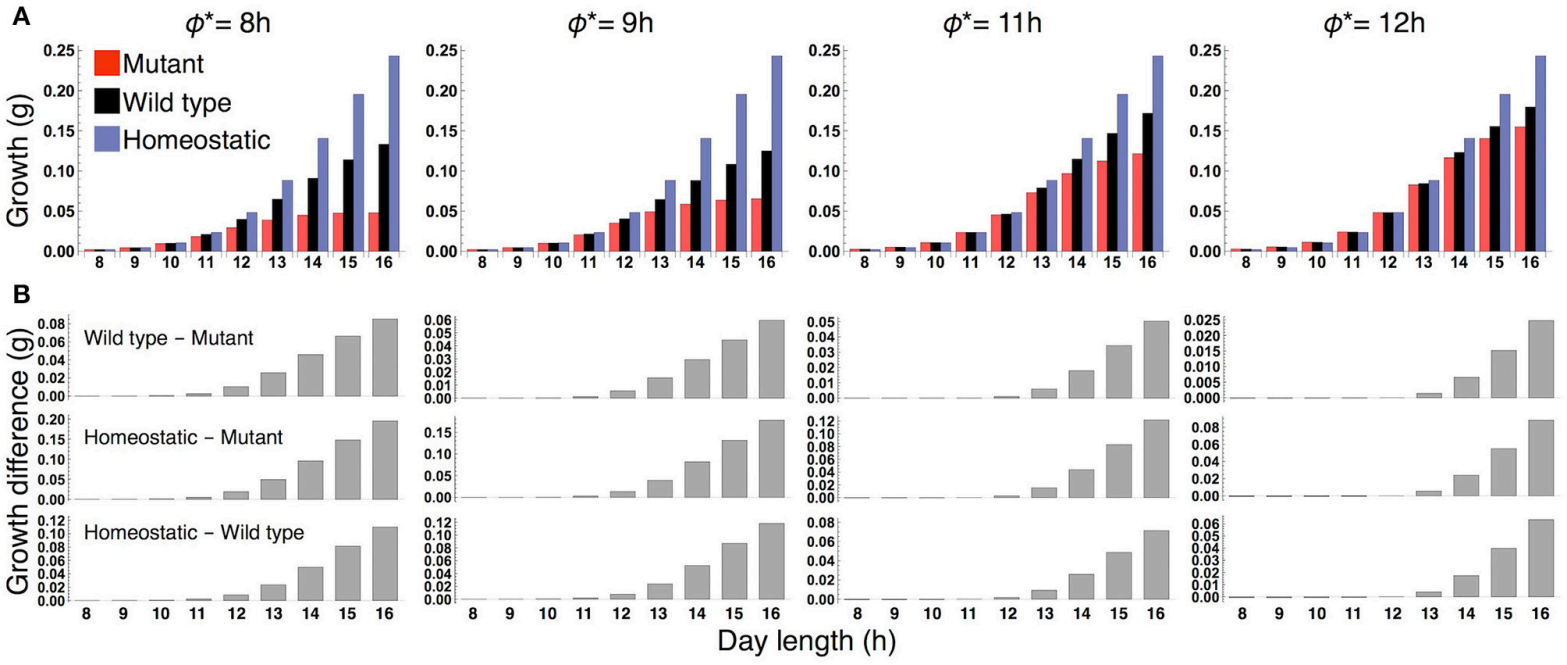
**(A)** 10-day growth of the sink (SAM or RAM) of the mutant, wild type, and homeostatic plant, and **(B)** growth differences among the plants at various values of the subjective dusk ϕ^*^.

**Figure 7:**
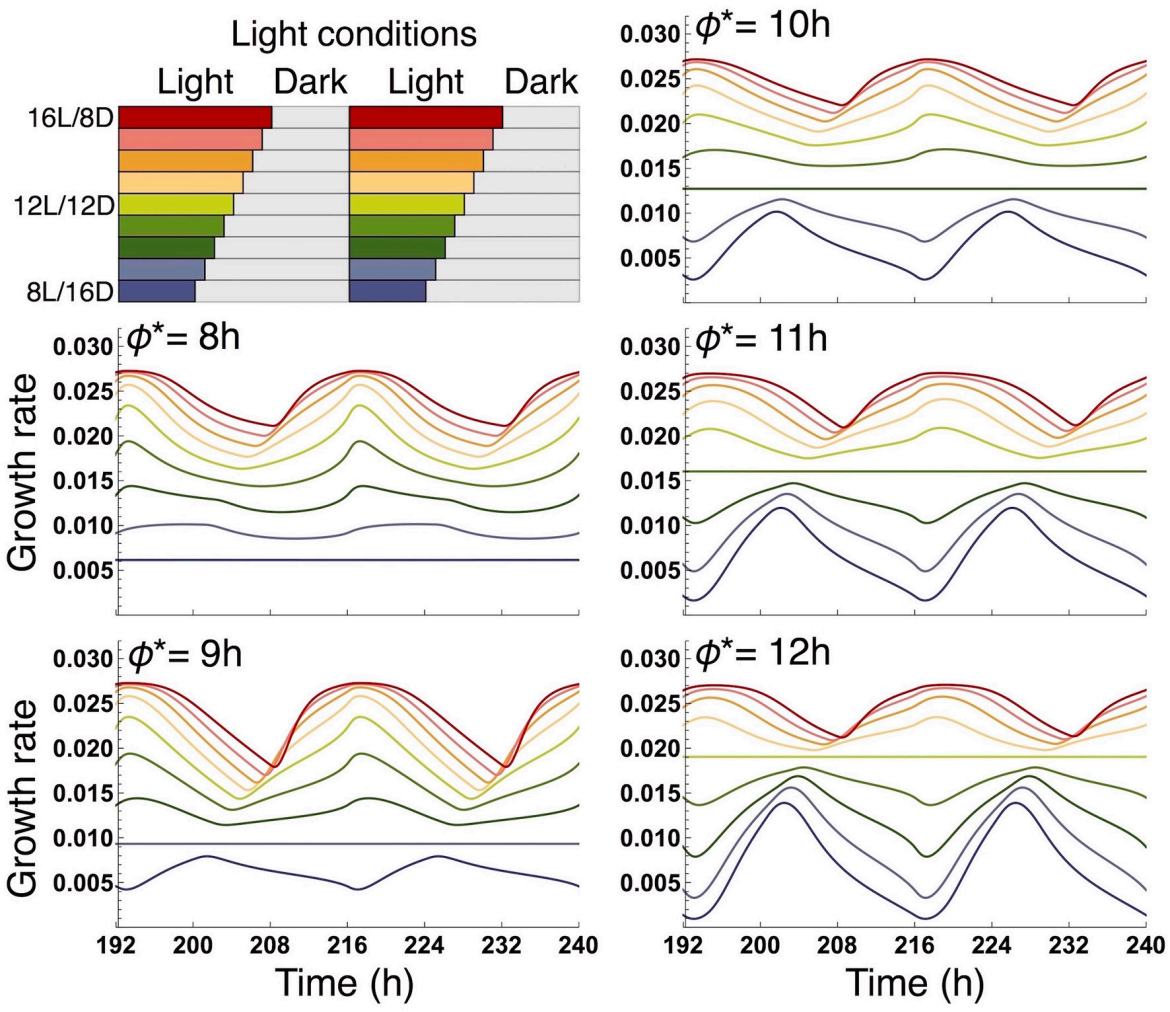
Comparison of growth rate of the sink (SAM or RAM) of the wild type at various values of the subjective dusk ϕ^*^. In each panel, growth dynamics in various photoperiods is represented. Different colors correspond to different light conditions (upper left panel).

We examined the effect of the timing of the subjective dusk ϕ^*^ on growth dynamics of the wild type (Figures 6, 7). Changes in ϕ^*^ do not markedly alter the values of 10-day growth of the sink and its difference among plant types (Figure 6). On the other hand, growth rate is strongly dependent on the timing of both the internal and external dusk. When the external dusk occurs later than the internal dusk (i.e., the length of the light period τ_L_ is larger than ϕ^*^), the growth rate peaks around dawn and decreases around dusk (Figures 4C, 7). As the photoperiod is shortened, amplitude of growth rate decreases and eventually becomes almost zero. When the value of τ_L_ is smaller than ϕ^*^, the opposite growth pattern is observed, with a peak around dusk and a trough around dawn (Figures 5C, 7).

**Figure 8:**
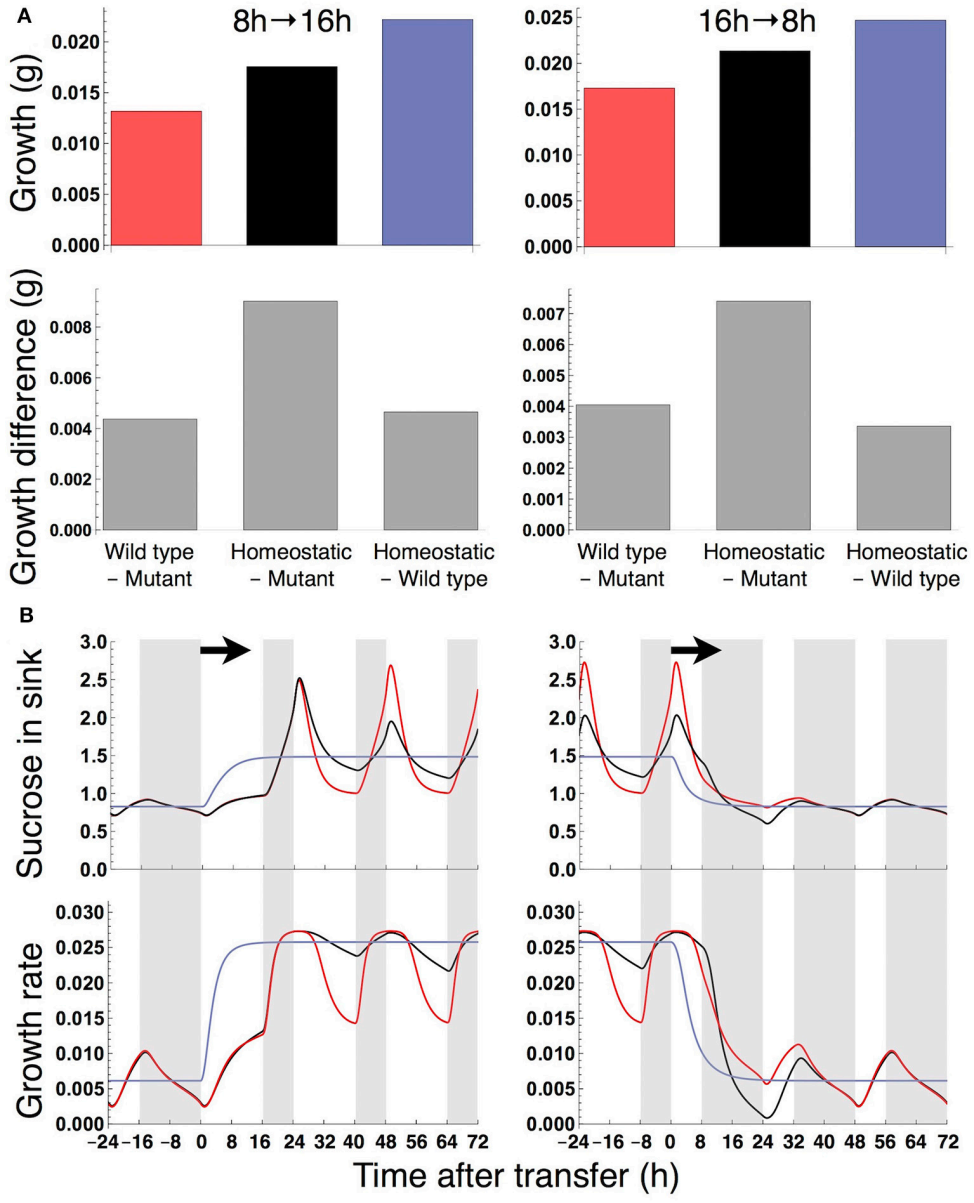
**(A)** Growth of the sink (SAM or RAM) of the mutant, wild type, and homeostatic plant transferred from an 8-h to 16-h photoperiod (left) and from a 16-h to 8-h photoperiod (right) and the growth difference among the plants. Color codes as in Figure 3A. **(B)** Predicted profiles of sucrose in the sink and growth rate around the photoperiod change. Details are as in the legend to Figure 4.

When the plants are transferred from long to short or short to long days, the growth of the wild type and homeostatic plant are consistently higher than that of the sugar-insensitive mutant (Figure 8A). All three plants are able to restore normal sugar and growth dynamics in about 2 days after the transfer, although the dynamics are complex immediately after the photoperiod change (Figure 8B). Therefore, the growth differences under these conditions may reflect the results under the constant photoperiod conditions that growth of the mutant is inferior to the others especially under a long day (Figure 3). Note that the dynamics of other variables such as sucrose in source were reported in a previous study (Seki et al., 2017).

## Discussion

Our results provide the first theoretical evidence that clock entrainment by photosynthetic products improves the fitness of higher plants. The sugar-sensitive wild type is predicted to grow faster than the sugar-insensitive mutant under long day conditions (Figure 3) because the phase shift of the circadian oscillator by sugar signals enables efficient sugar allocation for growth, while the mutant accumulates carbon as insoluble starch unusable for growth. The growth of the wild type is also higher than the mutant under changing photoperiod conditions (Figure 8A). Since the wild type and mutant are assumed to possess the same entrainment property to light, the lower growth in the mutant stems solely from the lack of sugar-induced phase adjustment. These results suggest that clock entrainment by endogenous sugar signals, in addition to entrainment by exogenous light signals, optimizes plant growth in nature, where the day length gradually changes.

Our results also provide important information about the internal timing of plants. Our model predicts that the growth pattern of the wild type displays a maximum around dawn and a minimum around dusk in a 16-h photoperiod (Figure 4C) or more generally under conditions where the value of τ_L_ (length of the light period) is larger than ϕ^*^ (timing of the subjective dusk) (Figure 7). When the value of τ_L_ is smaller than ϕ^*^, the pattern is reversed (Figure 5C where τ_L_ = 8 h and ϕ^*^ = 10 h; Figure 7). Yazdanbakhsh et al. (2011) reported, in both 16-h and 8-h photoperiods, a similar diel pattern in root elongation growth of *A. thaliana* (Col-0), which possibly correlates with fresh biomass change since both growth measures consider dry matter production and water content. These findings imply that ϕ^*^ of this accession is about 8 h. This idea is consistent with the PRC of the same accession to a sucrose pulse showing a transition from phase advance to delay, which corresponds to the phase ϕ^*^, at 6–10 h zeitgeber time (Haydon et al., 2013; Seki et al., 2017). These data suggest that a relatively short light period is subjectively anticipated by this accession. Other ecotypes of *A. thaliana* that anticipate later subjective dusk (i.e., large ϕ^*^) will display the inversed growth pattern in a short photoperiod and later phase advance-to-delay transition of the sucrose-pulsed PRC.

Numerous mathematical models on growth of various plants have been developed (e.g., Thornley and Johnson, 1990; Chew et al., 2014, 2017; Feller et al., 2015; Barillot et al., 2016a,b). Among them, two multiscale models of *A. thaliana* include the circadian clock sub-model and are able to quantitatively predict growth (Chew et al., 2014, 2017). Because the phase of the clock is dynamically regulated by sugar signals in our model and such phase adjustment is not considered in the previous models (Chew et al., 2014, 2017), only our model enables us to evaluate the impact of phase regulation by sugar on plant growth. Our model correctly predicts the empirical finding that wild type *A. thaliana* (Col-0) and the mutant *prr7–11* in which phase response to sugar is abolished grow similarly in a 12-h photoperiod (Chew et al., 2017; Figure 3). We suggest that this is due to the relatively weak impact of sugar entrainment on growth, as our modeling results show that the sugar-induced phase shift has less effect on growth dynamics in shorter photoperiods (Figure 5). These considerations are reminiscent of the empirical finding that response of *A. thaliana* to induced increases in trehalose-6-phosphate, a potential signal metabolite of sucrose status, was more intense in a 16-h photoperiod than in a 12-h photoperiod (Figueroa et al., 2016). Our model also predicts significantly faster growth of the wild type than the sugar-insensitive mutant under long day conditions (Figure 3), which will be experimentally tested using wild type *A. thaliana* and *prr7–11*. Wild type is likely to grow faster than *prr7–11* under long day conditions because the latter accumulates more starch than the former in a 16-h photoperiod (Seki et al., 2017) which implies that *prr7–11* can utilize less sucrose for growth than wild type. However, it should be noted that mutation of *PRR7* affects not only sugar signaling but also light signaling in the plant circadian system (Kaczorowski and Quail, 2003; Farré et al., 2005), and thus the growth characteristics of *prr7–11* can be influenced by variations in light as well as sugar entrainment. Nevertheless, sugar entrainment is likely to be a dominant factor because our previous study has theoretically shown that the deficient in sugar signaling only is sufficient to correctly predict patterns of starch turnover of *prr7–11* (Seki et al., 2017).

There are several possibilities to expand our model. We used a constant rate *a* for carbon capture, which was determined in a previous study based on experiments where nutrient levels were controlled (Gibon et al., 2004; Feugier and Satake, 2013). In higher plants, however, it has been demonstrated that sugar accumulation downregulates photosynthesis, possibly through decreased activity of Rubisco (Araya et al., 2006; Ribeiro et al., 2012; Quentin et al., 2013; Lobo et al., 2015). Formalizing the carbon capture rate as a decreasing function of sucrose concentration could reduce the growth of the sugar-insensitive mutant under long day conditions due to the very high sucrose level around dawn (Figures 4A,B). Regarding circadian clock properties, we considered a circadian oscillator with a period of 24 h (i.e., ω = 1). A recent study has reported a positive correlation between the free-running period of natural populations of *Mimulus guttatus* and the latitude of their geographic origin (Greenham et al., 2017). In *A. thaliana*, a similar but weak correlation has been found (Michael et al., 2003), although advantages of such variation in the period remain elusive. Growth simulations of plants with fast- and slow-running clocks could potentially reveal the adaptive value of natural variation in the circadian period in terms of growth optimization. Moreover, we can consider the possibility that two or more clock genes are involved in the phase response to sugar, where each gene can have differential responsiveness. Although the crucial role of *PRR7* in the response has been established (Haydon et al., 2013), other clock genes could participate in it; for instance, *CCA1* will be a good candidate, mutation of which disrupts the dependency of the circadian period on sucrose concentration in growth media (Haydon et al., 2013). To examine this possibility, the single phase oscillator will be expanded to coupled two or multiple oscillators model, in which each phase oscillator is defined with different PRC to sucrose and sugar input function (*Z*_S_ and *f*_S_ in Equation (4), respectively). Alternative way is to use the clock gene-regulatory network model (Fogelmark and Troein, 2014; De Caluwé et al., 2016) with the explicit formalization of the interaction between clock genes and sugar signals.

We conclude that plants optimize growth by monitoring nutrient status and utilizing endogenous sugar signals as circadian-phase regulators. Photosynthetic products act not only as direct growth substances but also as feedback signals to the clock to realize the efficient carbon-usage for growth. It is plausible that plants as sessile organisms utilize signals from metabolism because they seem more controllable than environmental signals. In this sense, the phase regulation by endogenous signals is potentially even more important for plants than animals that also utilize metabolic signals as zeitgebers (Woller et al., 2016). Our data strongly support the concept that the circadian clock improves the fitness of organisms by forming complex feedback loops with the signaling pathways it controls (Sanchez and Kay, 2016).

## Author contributions

TO and AS developed the model and wrote the manuscript. TO analyzed the model.

### Conflict of interest statement

The authors declare that the research was conducted in the absence of any commercial or financial relationships that could be construed as a potential conflict of interest.

## References

[B1] Araya T. Noguchi K. Terashima I. (2006). Effects of carbohydrate accumulation on photosynthesis differ between sink and source leaves of *Phaseolus vulgaris* L. Plant Cell Physiol. 47, 644–652. 10.1093/pcp/pcj033 16540483

[B2] Baena-González E. Sheen J. (2008). Convergent energy and stress signaling. Trends Plant Sci. 13, 474–482. 10.1016/j.tplants.2008.06.006 18701338 PMC3075853

[B3] Barillot R. Chambon C. Andrieu B. (2016a). CN-Wheat, a functional–structural model of carbon and nitrogen metabolism in wheat culms after anthesis. I. Model description. Ann. Bot. 118, 997–1013. 10.1093/aob/mcw143 PMC5055822 27497242

[B4] Barillot R. Chambon C. Andrieu B. (2016b). CN-Wheat, a functional–structural model of carbon and nitrogen metabolism in wheat culms after anthesis. II. Model evaluation. Ann. Bot. 118, 1015–1031. 10.1093/aob/mcw144 PMC5055823 27497243

[B5] Caspar T. Huber S. C. Somerville C. (1985). Alterations in growth, photosynthesis, and respiration in a starchless mutant of *Arabidopsis thaliana* (L.) deficient in chloroplast phosphoglucomutase activity. Plant Physiol. 79, 11–17. 10.1104/pp.79.1.11 16664354 PMC1074821

[B6] Chew Y. H. Seaton D. D. Mengin V. Flis A. Mugford S. T. Smith A. M. (2017). Linking circadian time to growth rate quantitatively via carbon metabolism. Biorxiv 10.1101/105437

[B7] Chew Y. H. Wenden B. Flis A. Mengin V. Taylor J. Davey C. L.. (2014). Multiscale digital Arabidopsis predicts individual organ and whole-organism growth. Proc. Natl. Acad. Sci. U.S.A. 111, 4127–4136. 10.1073/pnas.1410238111 25197087 PMC4191812

[B8] Christophe A. Letort V. Hummel I. Coumède P.-H. de Reffye P. Lecoeur J. (2008). A model-based analysis of the dynamics of carbon balance at the whole-plant level in *Arabidopsis thaliana*. Funct. Plant Biol. 35, 1147–1162. 10.1071/FP08099 32688862

[B9] De Caluwé J. Xiao Q. Hermans C. Verbruggen N. Leloup J.-C. Gonze D. (2016). A compact model for the complex plant circadian clock. Front. Plant Sci. 7:74. 10.3389/fpls.2016.00074 26904049 PMC4742534

[B10] Deprost D. Yao L. Sormani R. Moreau M. Leterreux G. Nicolai M.. (2007). The Arabidopsis TOR kinase links plant growth, yield, stress resistance and mRNA translation. EMBO Rep. 8, 864–870. 10.1038/sj.embor.7401043 17721444 PMC1973950

[B11] Dodd A. N. Salathia N. Hall A. Kévei E. Tóth R. Nagy F.. (2005). Plant circadian clocks increase photosynthesis, growth, survival, and competitive advantage. Science 309, 630–633. 10.1126/science.1115581 16040710

[B12] Farré E. M. Harmer S. L. Harmon F. G. Yanovsky M. J. Kay S. A. (2005). Overlapping and distinct roles of PRR7 and PRR9 in the *Arabidopsis circadian* clock. Curr. Biol. 15, 47–54. 10.1016/j.cub.2004.12.067 15649364

[B13] Feller C. Favre P. Janka A. Zeeman S. C. Gabriel J.-P. Reinhardt D. (2015). Mathematical modeling of the dynamics of shoot-root interactions and resource partitioning in plant growth. PLoS ONE 10:e0127905. 10.1371/journal.pone.0127905 26154262 PMC4495989

[B14] Feugier F. G. Satake A. (2013). Dynamical feedback between circadian clock and sucrose availability explains adaptive response of starch metabolism to various photoperiods. Front. Plant Sci. 3:305. 10.3389/fpls.2012.00305 23335931 PMC3544190

[B15] Figueroa C. M. Feil R. Ishihara H. Watanabe M. Kölling K. Krause U.. (2016). Trehalose 6–phosphate coordinates organic and amino acid metabolism with carbon availability. Plant J. 85, 410–423. 10.1111/tpj.13114 26714615

[B16] Fogelmark K. Troein C. (2014). Rethinking transcriptional activation in the *Arabidopsis circadian* clock. PLoS Comput. Biol. 10:e1003705. 10.1371/journal.pcbi.1003705 25033214 PMC4102396

[B17] Gibon Y. Bläsing O. E. Palacios-Rojas N. Pankovic D. Hendriks J. H. M. Fisahn J.. (2004). Adjustment of diurnal starch turnover to short days: depletion of sugar during the night leads to a temporary inhibition of carbohydrate utilization, accumulation of sugars and post-translational activation of ADP-glucose pyrophosphorylase in the following light period. Plant J. 39, 847–862. 10.1111/j.1365-313X.2004.02173.x 15341628

[B18] Graf A. Smith A. M. (2011). Starch and the clock: the dark side of plant productivity. Trends Plant Sci. 16, 169–175. 10.1016/j.tplants.2010.12.003 21216654

[B19] Graf A. Schlereth A. Stitt M. Smith A. M. (2010). Circadian control of carbohydrate availability for growth in Arabidopsis plants at night. Proc. Natl. Acad. Sci. U.S.A. 107, 9458–9463. 10.1073/pnas.0914299107 20439704 PMC2889127

[B20] Greenham K. Lou P. Puzey J. R. Kumar G. Arnevik C. Farid H.. (2017). Geographic variation of plant circadian clock function in natural and agricultural settings. J. Biol. Rhythms 32, 26–34. 10.1177/0748730416679307 27920227

[B21] Haydon M. J. Mielczarek O. Robertson F. C. Hubbard K. E. Webb A. A. R. (2013). Photosynthetic entrainment of the *Arabidopsis thaliana* circadian clock. Nature 502, 689–692. 10.1038/nature12603 24153186 PMC3827739

[B22] Johnson C. H. Elliott J. A. Foster R. (2003). Entrainment of circadian programs. Chronobiol. Int. 20, 741–774. 10.1081/CBI-120024211 14535352

[B23] Kaczorowski K. A. Quail P. H. (2003). Arabidopsis pseudo-response regulator7 is a signaling intermediate in phytochrome-regulated seedling deetiolation and phasing of the circadian clock. Plant Cell 15, 2654–2665. 10.1105/tpc.015065 14563930 PMC280569

[B24] Lastdrager J. Hanson J. Smeekens S. (2014). Sugar signals and the control of plant growth and development. J. Exp. Bot. 65, 799–807. 10.1093/jxb/ert474 24453229

[B25] Lobo A. K. M. Martins M. O. Neto M. C. L. Machado E. C. Ribeiro R. V. Silveira J. A. G. (2015). Exogenous sucrose supply changes sugar metabolism and reduces photosynthesis of sugarcane through the down-regulation of Rubisco abundance and activity. J. Plant Physiol. 179, 113–121. 10.1016/j.jplph.2015.03.007 25863283

[B26] Locke J. C. W. Southern M. M. Kozma-Bognár L. Hibberd V. Brown P. E. Turner M. S.. (2005). Extension of a genetic network model by iterative experimentation and mathematical analysis. Mol. Syst. Biol. 1:0013. 10.1038/msb4100018 16729048 PMC1681447

[B27] Lu Y. Gehan J. P. Sharkey T. D. (2005). Daylength and circadian effects on starch degradation and maltose metabolism. Plant Physiol. 138, 2280–2291. 10.1104/pp.105.061903 16055686 PMC1183414

[B28] Mengin V. Pyl E.-T. Moraes T. A. Sulpice R. Krohn N. Encke B.. (2017). Photosynthate partitioning to starch in *Arabidopsis thaliana* is insensitive to light intensity but sensitive to photoperiod due to a restriction on growth in the light in short photoperiods. Plant Cell Environ. 40, 2608–2627. 10.1111/pce.13000 28628949

[B29] Michael T. P. Salomé P. A. Yu H. J. Spencer T. R. Sharp E. L. McPeek M. A.. (2003). Enhanced fitness conferred by naturally occurring variation in the circadian clock. Science 302, 1049–1053. 10.1126/science.1082971 14605371

[B30] Münch E. (1930). Die Stoffbewegung in der Pflanze. Jena: Fischer.

[B31] Quentin A. G. Close D. C. Hennen L. M. H. P. Pinkard E. A. (2013). Down-regulation of photosynthesis following girdling, but contrasting effects on fruit set and retention, in two sweet cherry cultivars. Plant Physiol. Biochem. 73, 359–367. 10.1016/j.plaphy.2013.10.014 24189522

[B32] Ribeiro R. V. Machado E. C. Habermann G. Santos M. G. Oliveira R. F. (2012). Seasonal effects on the relationship between photosynthesis and leaf carbohydrates in orange trees. Funct. Plant Biol. 39, 471–480. 10.1071/FP11277 32480798

[B33] Sanchez S. E. Kay S. A. (2016). The plant circadian clock: from a simple timekeeper to a complex developmental manager. Cold Spring Harbor Perspect. Biol. 8:a027748. 10.1101/cshperspect.a027748 27663772 PMC5131769

[B34] Satake A. Seki M. Iima M. Teramoto T. Nishiura Y. (2016). Florigen distribution determined by a source-sink balance explains the diversity of inflorescence structures in Arabidopsis. J. Theor. Biol. 395, 227–237. 10.1016/j.jtbi.2016.01.035 26845309

[B35] Scialdone A. Mugford S. T. Feike D. Skeffington A. Borrill P. Graf A.. (2013). Arabidopsis plants perform arithmetic division to prevent starvation at night. Elife 2:e00669. 10.7554/eLife.00669 23805380 PMC3691572

[B36] Seki M. Feugier F. G. Song X.-J. Ashikari M. Nakamura H. Ishiyama K.. (2015). A mathematical model of phloem sucrose transport as a new tool for designing rice panicle structure for high grain yield. Plant Cell Physiol. 56, 605–619. 10.1093/pcp/pcu191 25516572

[B37] Seki M. Ohara T. Hearn T. J. Frank A. da Silva V. C. H. Caldana C. (2017). Adjustment of the *Arabidopsis circadian* oscillator by sugar signaling dictates the regulation of starch metabolism. Sci. Rep. 7:8305 10.1038/s41598-017-08325-y 28814797 PMC5559614

[B38] Smith A. M. Stitt M. (2007). Coordination of carbon supply and plant growth. Plant Cell Environ. 30, 1126–1149. 10.1111/j.1365-3040.2007.01708.x 17661751

[B39] Smith S. M. Fulton D. C. Chia T. Thorneycroft D. Chapple A. Dunstan H.. (2004). Diurnal changes in the transcriptome encoding enzymes of starch metabolism provide evidence for both transcriptional and posttranscriptional regulation of starch metabolism in Arabidopsis leaves. Plant Physiol. 136, 2687–2699. 10.1104/pp.104.044347 15347792 PMC523333

[B40] Stitt M. Zeeman S. C. (2012). Starch turnover: pathways, regulation and role in growth. Curr. Opin. Plant Biol. 15, 282–292. 10.1016/j.pbi.2012.03.016 22541711

[B41] Sulpice R. Flis A. Ivakov A. A. Apelt F. Krohn N. Encke B.. (2014). Arabidopsis coordinates the diurnal regulation of carbon allocation and growth across a wide range of photoperiods. Mol. Plant 7, 137–155. 10.1093/mp/sst127 24121291

[B42] Thornley J. H. M. Johnson I. R. (1990). Plant and Crop Modeling: A Mathematical Approach to Plant and Crop Physiology. Oxford: Clarendon Press.

[B43] Woelfle M. A. Ouyang Y. Phanvijhitsiri K. Johnson C. H. (2004). The adaptive value of circadian clocks: an experimental assessment in cyanobacteria. Curr. Biol. 14, 1481–1486. 10.1016/j.cub.2004.08.023 15324665

[B44] Woller A. Duez H. Staels B. Lefranc M. (2016). A mathematical model of the liver circadian clock linking feeding and fasting cycles to clock function. Cell Rep. 17, 1087–1097. 10.1016/j.celrep.2016.09.060 27760313

[B45] Yazdanbakhsh N. Sulpice R. Graf A. Stitt M. Fisahn J. (2011). Circadian control of root elongation and C partitioning in *Arabidopsis thaliana*. Plant Cell Environ. 34, 877–894. 10.1111/j.1365-3040.2011.02286.x 21332506

[B46] Younginger B. S. Sirová D. Cruzan M. B. Ballhorn D. J. (2017). Is biomass a reliable estimate of plant fitness? Appl. Plant Sci. 5:1600094. 10.3732/apps.1600094 28224055 PMC5315378

