# Supplementary material for: Photosynthetic Entrainment of the Circadian Clock Facilitates Plant Growth under Environmental Fluctuations: Perspectives from an Integrated Model of Phase Oscillator and Phloem Transportation
Source: Front Plant Sci. 2017 Oct 30;8:1859. doi: 10.3389/fpls.2017.01859 (PMC5670358; doi:10.3389/fpls.2017.01859)
Supplement: Supplementary file 1 [file Presentation1.pdf]

Supplementary Material

Photosynthetic entrainment of the circadian clock facilitates plant growth under environmental fluctuations: perspectives from an integrated model of phase oscillator and phloem transportation

Takayuki Ohara*, Akiko Satake

*** Correspondence:** Takayuki Ohara: t.ohara.63920@ees.hokudai.ac.jp

1 Sucrose solution flux through phloem tubes

Fluxes of sucrose solution and pure water were modeled as in previous studies that addressed the phloem transport of sucrose (Seki et al., 2015) or a flowering signal (florigen) (Satake et al., 2016). Modeling was based on the pressure-flow hypothesis, which states that the difference in hydrostatic pressure between the source and sink generates the solution flux (Münch, 1930). We assume that sucrose concentration is uniform within each tube, an approximation used in the previous studies to predict the realistic grain arrangement of rice (Seki et al., 2015) and various inflorescence structures observed in *Arabidopsis thaliana* mutants (Satake et al., 2016). The sucrose concentration in tube i ($i \in \{0, ..., 5\}$; Fig. 1C) at time t is designated as $g_i(t)$. The phloem tubes are represented as rigid cylinders (Figs. 1A, B). The sets of the source leaf, tubes, and sink tissues are represented by symbols **G**, **T**, and **Y**, respectively, where $\mathbf{G} = \{0\}$, $\mathbf{T} = \{1, 2, 3\}$, and $\mathbf{Y} = \{4, 5\}$ (Figs. 1A-C).

1.1 Sucrose flux as Hagen–Poiseuille flow

Inertia of phloem sap flow is considered negligibly small compared to its viscosity (Thompson, 2006); thus, sucrose solution flux $J_i(t)$ can be calculated using the Hagen–Poiseuille equation:

$$J_i(t) = \frac{\pi}{8\nu}\frac{r_i^4}{l_i}(p_k(t) - p_i(t)), \tag{S1}$$

where ν is the viscosity of phloem sap. r_i and l_i are the radius and length of component i, respectively, and correspond to r_T and l_T ($i \in \mathbf{T}$) and r_Y and l_Y ($i \in \mathbf{Y}$) (Fig. 1B). $p_k(t)$ and $p_i(t)$ are the turgor pressures at the apex of component k and i, respectively (Fig. 1C). The turgor pressures are caused by the pure water flow between phloem and xylem, which is detailed below. We define the direction of the phloem sap so that it flows from component k to i when J_i(t) is positive, while it flows from component i to k when J_i(t) is negative.

1.2 Phloem-xylem flow of pure water

Pure water flows from (or toward) the phloem tube toward (or from) xylem due to osmosis, which is assumed to occur at the small side surface near the apex of each component (Fig. 1D). Water flux

$w_i(t)$ depends on the difference between the turgor pressure and the osmotic pressure:

$$w_i(t) = mA_i(p_i(t) - g_i(t)RT), \quad \text{(S2)}$$

where m is per-area permeability, and A_i is the surface area of component i. Under the assumption of dilute solution, the osmotic pressure is calculated as $g_i(t)RT$ by the van't Hoff equation, where R and T are the gas constant and absolute temperature, respectively.

Because we assume that every cylinder is rigid, water efflux should be equal to water influx at each component (Fig. 1D). The conservation law of water volume is therefore held:

$$0 = J_1(t) + w_0(t), \quad \text{(S3)}$$

$$J_1(t) = J_2(t) + J_3(t) + w_1(t), \quad \text{(S4)}$$

$$J_i(t) = J_{i+2}(t) + w_i(t) \ \ (i \in \mathbf{T} \setminus \{1\}), \quad \text{(S5)}$$

$$J_i(t) = w_i(t) \ \ (i \in \mathbf{Y}), \quad \text{(S6)}$$

Turgor pressure $p_i(t)$ is algebraically represented by solving a set of simultaneous linear equations (Eqs. (S1)-(S6)). $p_i(t)$ depends solely on the sucrose concentration in the phloem, the dynamics of which is formalized below. Sucrose flux $J_i(t)$ is then calculated by substituting $p_i(t)$ into Eq. (S1).

1.3 Sucrose dynamics in the phloem tube

To describe the sucrose dynamics in the phloem, we introduce the symbol $[x]_+$ meaning $\max\{0, x\}$. Sucrose in component k is transported to component i when J_i(t) is positive. The amount of sucrose transported from k to i per unit time is given by $g_k(t)J_i(t)$. In contrast, when $J_i(t)$ is negative, sucrose is transported from component i to k at an amount given by $g_i(t)(-J_i(t))$. These two cases are integrated into the term $g_k(t)[J_i(t)]_+ - g_i(t)[-J_i(t)]_+$ (respectively, $-g_k(t)[J_i(t)]_+ + g_i(t)[-J_i(t)]_+$), which denotes the rate of the sucrose change due to the flux $J_i(t)$ at component i (respectively, component k). By applying this consideration to each component, sucrose dynamics in the phloem are described by the following equations:

$$\frac{d}{dt}g_1(t) = \frac{1}{V_1}\{g_0(t)[J_1(t)]_+ + g_2(t)[-J_2(t)]_+ + g_3(t)[-J_3(t)]_+ - g_1(t)([-J_1(t)]_+ + [J_2(t)]_+ + [J_3(t)]_+)\}, \quad \text{(S7)}$$

$$\frac{d}{dt}g_i(t) = \frac{1}{V_i}\{g_1(t)[J_i(t)]_+ + g_{i+2}(t)[-J_{i+2}(t)]_+ - g_i(t)([-J_i(t)]_+ + [J_{i+2}(t)]_+)\} \ \ (i \in \mathbf{T} \setminus \{1\}), \quad \text{(S8)}$$

where V_i represents the volume of the source ($i \in \mathbf{G}$), the tubes ($i \in \mathbf{T}$), and the sinks ($i \in \mathbf{Y}$). The dynamics of $g_i(t)$ (i = 0, 4, and 5) are described by Eqs. (3) and (5) in the main text.

2 Parameter estimation of the sugar input function

Similar to a previous study (Seki et al., 2017), we define the sugar input function f_S by the Hill equation:

$$f_S(\tilde{S}_G) = \mathrm{sgn}(\tilde{S}_G)\frac{|\tilde{S}_G|^n}{K^n + |\tilde{S}_G|^n}, \tag{S9}$$

where sgn is a sign function, and n and K are constants. The parameter values of K and n are determined for various values of the subjective dusk φ^* using the procedure described in a previous study (Seki et al., 2017).

3 Alternative formalization of growth rate

In the main text, we define growth rate $\lambda\alpha(S_Y)$ of the sink tissues as a nonlinear function of sucrose concentration (Eqs. (7) and (8)). Here we test the alternative formalization of growth rate as a linear function of sucrose. We assume that sucrose is consumed for growth at a rate α_1 and that growth is repressed when sucrose falls below a threshold level. Based on these assumptions, the dynamics of sucrose S_Y and plant fresh weight W_Y are described by

$$\frac{d}{dt}S_Y(t) = \eta_Y g_i(t) - h_Y S_Y(t) - \alpha_1 S_Y(t), \tag{S10}$$

$$\frac{d}{dt}W_Y(t) = \lambda(\alpha_1 S_Y - \alpha_2[S_Y^* - S_Y]_+)W_Y(t), \tag{S11}$$

where α_2 is a parameter, S_Y^* is a threshold constant, and the symbol $[x]_+$ is defined as in Section 1.3 above. The term $\lambda(\alpha_1 S_Y - \alpha_2[S_Y^* - S_Y]_+)$ in Eq. (S11) is considered as the growth rate (Fig. S4A) and is referred to as "linear growth rate". The growth rate in the main text is correspondingly referred to as "nonlinear growth rate". When sucrose concentration is relatively low, the linear growth rate becomes negative (Fig. S4A), which corresponds to death of living tissues due to insufficient maintenance respiration. We estimated the values of α_1 and α_2 using procedures similar to those explained in Section 2.2.2 of the main text. We set $S_Y^* = 0.9$.

Similar to the results in the main text assuming the nonlinear growth with sucrose (Fig. 3), 10-day growth of the mutant is lower than the wild type and homeostatic plant under constant photoperiod conditions (Fig. S4B). However, the growth of all three plants in long photoperiods are closer using the linear growth rate than when computed using the nonlinear growth rate. Growth patterns are significantly altered by the change in formalization of the growth rate (Figs. S4C and D; Figs. 4C and 5C), while the sugar dynamics are not (data not shown). Under a long day (16 L/ 8 D) with the linear growth rate, the very high sucrose level around dawn (Fig. 4B) strongly contributes to growth of the mutant, in contrast to the case using the nonlinear growth rate (Fig. S4C; Fig. 4C), because growth rate keeps increasing with sucrose concentration. This reduces the growth difference between the mutant and the others. Under a short day (8 L/ 16 D) with the linear growth rate, the overall growth trend is unchanged, but its baseline is higher compared to that with the nonlinear growth rate (Fig. S4D; Fig.5C) simply because of the higher growth rate at a sucrose level of about 0.8 (Fig. S4A and Fig. 5B).

When the plants are transferred from a short to a long photoperiod, the growth of the wild type and homeostatic plant are still greater than the mutant (Fig. S5A) as in the case for the nonlinear growth model. However, the mutant grows faster than the others when the plants are transferred from a long to a short photoperiod (Fig. S5B). Transient dynamics of the growth rates immediately after the photoperiod change from short to long (Fig. S5C) are similar to those assuming the nonlinear growth rate (Fig. 8B). On the other hand, when photoperiod changes from long to short (Fig. S5D) the growth rate is transiently higher in the mutant (around $t = 0$), lower in the wild type (around $t = 24$), and almost the same in the homeostatic plant compared to the dynamics assuming the nonlinear growth rate (Fig. 8B). These changes in transient growth dynamics of the plants exposed to the long-to-short day transition, in addition to the small growth difference among the plants in constant conditions, distinguish the overall growth from those described in the main text (Fig. S5B and Fig. 8A).

4 Supplementary Figures

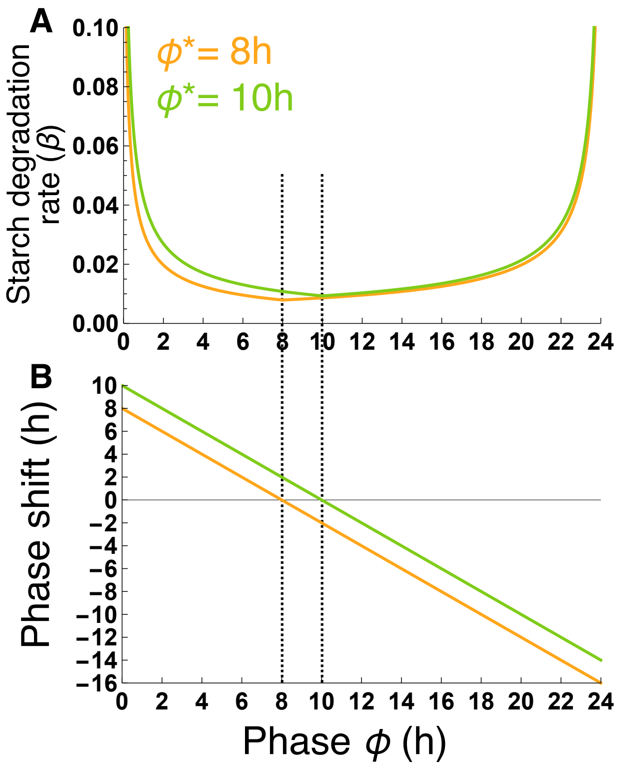

	Subjective dusk ϕ^*
Sugar-insensitive Mutant	Fixed
Wild type	Fixed
Homeostatic	Always equal to external photoperiod τ_L

Figure S1. (A) Starch degradation rate and **(B)** phase response curve to sucrose optimal for realizing sucrose homeostasis (Seki et al., 2017). Different colors correspond to different values of the subjective dusk φ^*. How φ^* is treated in the sugar-insensitive mutant, wild type, and homeostatic plant is summarized in the right table.

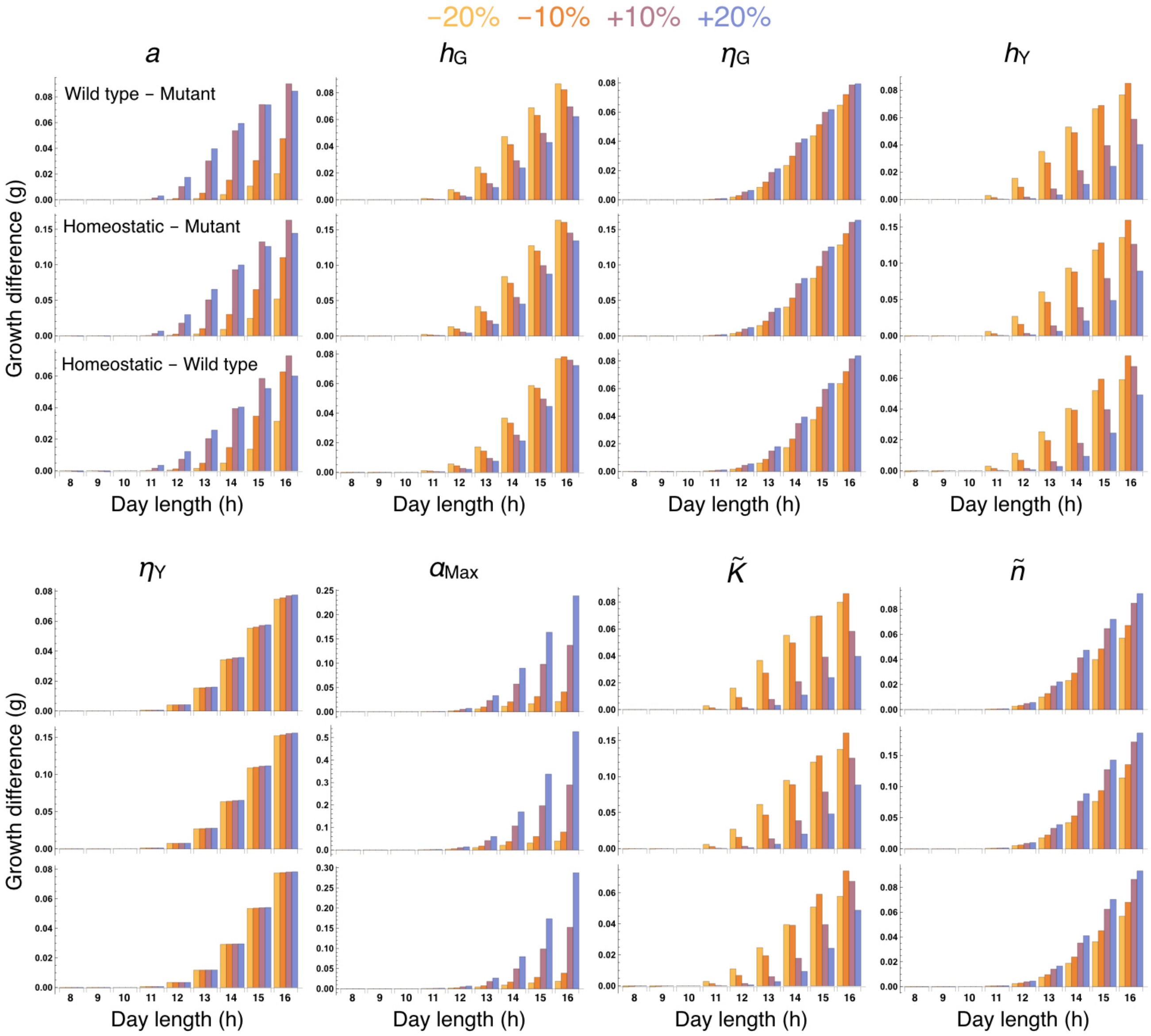

Figure S2. Growth difference among the mutant, wild type, and homeostatic plant under the change of parameter values. Changed parameters are represented above each group of three figures. Different colors correspond to the different extents of the parameter change shown at the top.

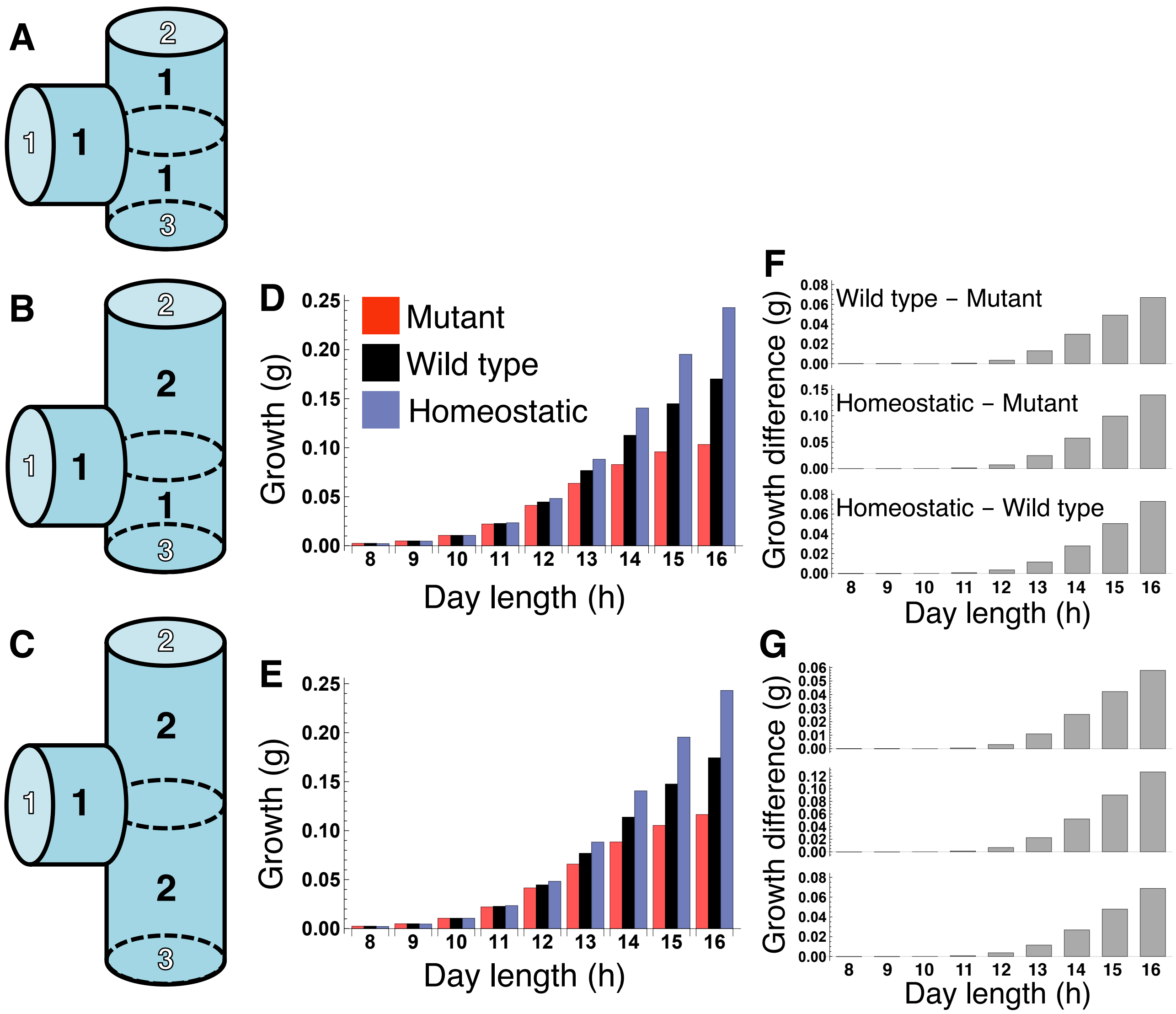

Figure S3. **(A)**, **(B)**, **(C)** Configurations of the phloem tubes used for computation of growth in the main text, **(D)**, and **(E)**, respectively. Black letters in each tube stand for the ratio of the tube lengths. **(D)**, **(E)** 10-day growth of the sink of the mutant, wild type, and homeostatic plant. **(F)**, **(G)** Growth difference among the plants based on the data in **(D)** and **(E)**, respectively. In **(D)** and **(F)**, the result for shoot apical meristem (SAM) only is shown because that of the root apical meristem (RAM) is almost the same, despite the length difference between tubes 2 and 3.

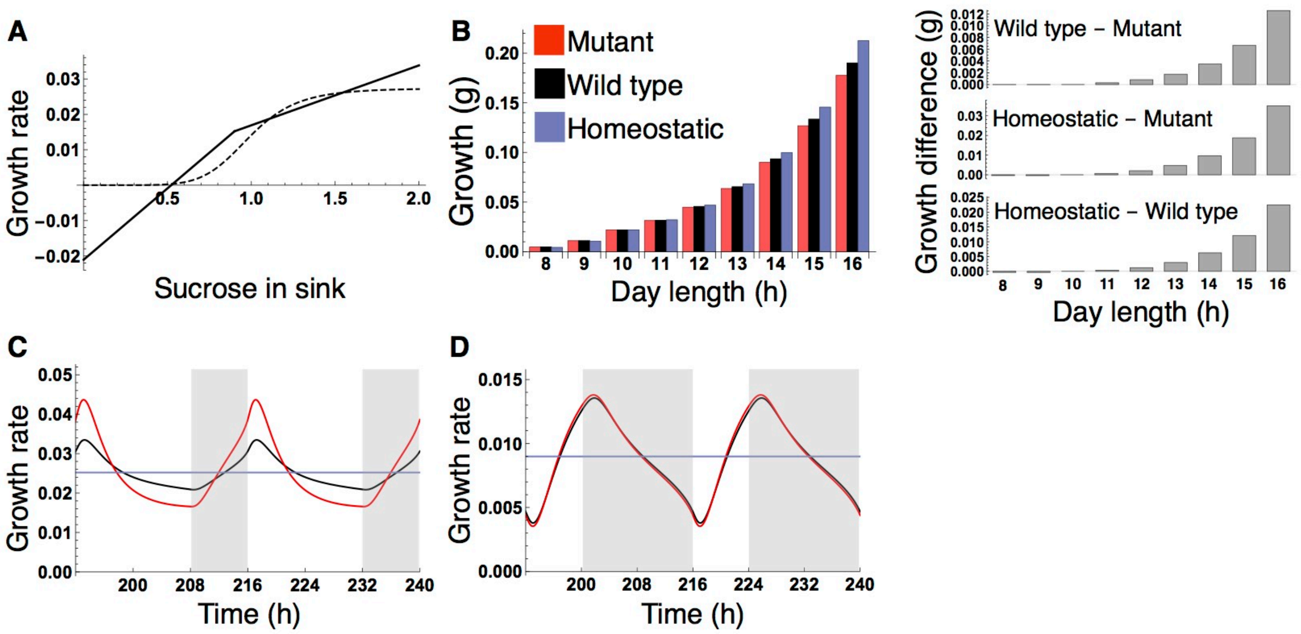

Figure S4. **(A)** Growth rate linearly dependent on sucrose (solid line). The growth rate formalized in the main text is also shown (dashed line). **(B)** 10-day growth of the sink (SAM or RAM) of the mutant, wild type, and homeostatic plant with the linear growth rate under constant photoperiod conditions, and the growth difference among the plants. **(C)**, **(D)** Predicted profiles of the linear growth rate of the plants in a 16-h **(C)** and 8-h **(D)** photoperiod. The unit for sucrose is μmolC_6 g^{-1}FW. White background: light period; Gray background: dark period.

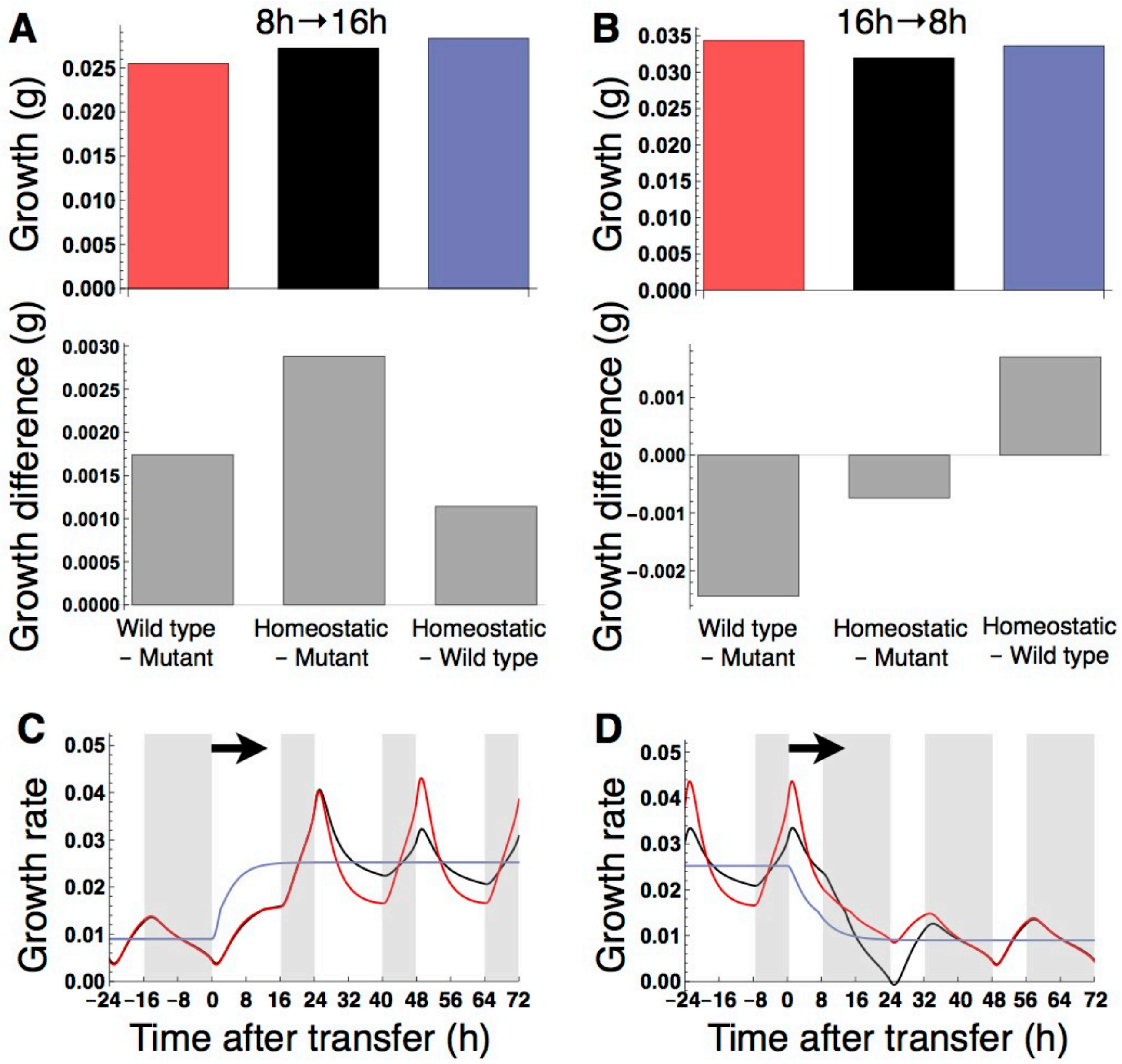

Figure S5. (A), **(B)** Growth of the sink (SAM or RAM) of the mutant, wild type, and homeostatic plant with the linear growth rate transferred from an 8-h to 16-h photoperiod **(A)** and from a 16-h to 8-h photoperiod **(B)** as well as the growth difference among the plants. Color codes as in Fig. S4B. **(C)**, **(D)** Predicted profiles of the linear growth rate around the photoperiod change. Details are as in the legend to Fig. S4.

5 Supplementary tables

Table S1. Summary of parameters, variables, and functions.

		Definition	Units	Value
Parameters	a	Carbon capture rate	μmolC6/gFW/hour	6 (Feugier and Satake, 2013)
	γ	Carbon partitioning rate for starch		0.68 (φ^* = 8h) (Seki et al., 2017)<br>0.64 (φ^* = 9h)<br>0.6 (φ^* = 10, 11, 12h) (Seki et al., 2017)
	κ	Constant (starch degradation occurs in proportion to C^{κ})		2/3 (Seki et al., 2017)
	h_G	Respiration rate in source	1/hour	0.79 (Feugier and Satake, 2013)
	h_Y	Respiration rate in sink	1/hour	0.79
	η_G	Sucrose loading rate	1/hour	1.98 (Feugier and Satake, 2013)
	η_Y	Sucrose unloading rate	m^3/hour	1.58×10^{-9} (Satake et al., 2016)
	ω	Angular frequency of the circadian clock		1 (Seki et al., 2017)
	φ^*	Timing of subjective dusk	Hour	
	τ_L	Photoperiod	Hour	
	K	Half saturation constant of f_S		0.1 (φ^* = 8h) (Seki et al., 2017)<br>0.1 (φ^* = 9h) †<br>0.1 (φ^* = 10h) (Seki et al., 2017)<br>0.5 (φ^* = 11h) †

				0.3 (φ^* = 12h) (Seki et al., 2017)
	n	Constant determining the shape of f_S		0.5 (φ^* = 8h) (Seki et al., 2017)<br>1.4 (φ^* = 9h) †<br>1.0 (φ^* = 10h) (Seki et al., 2017)<br>1.2 (φ^* = 11h) †<br>3.0 (φ^* = 12h) (Seki et al., 2017)
	α_{Max}	Saturation constant of α	μmolC6/gFW/hour	7.6×10^{-6} †
	$\tilde{K}$	Half saturation constant of α	μmolC6/gFW	9.9×10^{-2} †
	$\tilde{n}$	Constant determining the shape of α		6.9 †
	α_1	Constant in the linear growth rate	1/hour	0.17 †
	α_2	Constant in the linear growth rate	1/hour	0.23 †
	S_Y^*	Threshold constant in the linear growth rate	μmolC6/gFW	0.9
	λ	Conversion rate of sucrose to growth	1/μmolC6/gFW	1
	r_G	Cylinder radius of a tube in the source	m	1.0×10^{-4}
	r_T	Cylinder radius of a connecting tube	m	1.0×10^{-4}
	r_Y	Cylinder radius of a tube in the sink	m	1.0×10^{-5}
	l_G	Cylinder height of a tube in the source	m	1.0×10^{-2}
	l_T	Cylinder height of a connecting tube	m	3.0×10^{-2} or<br>6.0×10^{-2} (Fig. S3)
	l_Y	Cylinder height of a tube in the sink	m	2.0×10^{-2}
	V_X	Volume of cylinder of type X	m^3	$\pi r_X^2 l_X$
	A_X	Side surface area of cylinder of type X	m^2	$2\pi r_X l_X$

	v	Solution viscosity	Pa s	8.9×10^{-4} (Seki et al., 2015)
	m	Per area membrane permeability	m/Pa/s	5.0×10^{-14} (Seki et al., 2015)
	R	Gas constant	J/K/mol	8.31
	T	Air temperature	K	298
Variables	S_G	Sucrose concentration in the source	μmolC6/gFW	
	S_Y	Sucrose concentration in the sink	μmolC6/gFW	
	C	Starch concentration	μmolC6/gFW	
	φ	Phase of the circadian clock	Hour	
	W_Y	Fresh weight of the sink	g	$W_Y(0) = 5.0 \times 10^{-4}$
	g_i	Sucrose concentration of component i	μmolC6/gFW/m^3	
	p_i	Turgor pressure of component i	Pa	
Functions	L	Light condition (1 under light; 0 under dark)		
	β	Starch degradation rate	1/hour	
	Z_S	Continuous phase response curve to sugar signal		
	f_S	Transformation function of sucrose signal		
	α	Sucrose consumption rate for growth	μmolC6/gFW/hour	

Note: Parameters in general use (*e.g.*, gas constant) are represented in SI units for clarity, which are used in numerical simulations with conversion of the units. The values marked with † are estimated in the current study. The remaining values are arbitrarily chosen because of the lack of empirical data.

References

Feugier, F. G., and Satake, A. (2013) Dynamical feedback between circadian clock and sucrose availability explains adaptive response of starch metabolism to various photoperiods. *Front. Plant Sci.* 3:305. doi: 10.3389/fpls.2012.00305

Münch, E. (1930) Die Stoffbewegung in der Pflanze. Fischer, Jena. (in German).

Satake, A., Seki, M., Iima, M., Teramoto, T., and Nishiura, Y. (2016) Florigen distribution determined by a source-sink balance explains the diversity of inflorescence structures in *Arabidopsis*. *J. Theor. Biol.* 395, 227–237. doi: 10.1016/j.jtbi.2016.01.035

Seki, M., Feugier, F. G., Song, X.-J., Ashikari, M., Nakamura, H., Ishiyama, K., et al. (2015) A mathematical model of phloem sucrose transport as a new tool for designing rice panicle structure for high grain yield. *Plant Cell Physiol.* 56, 605–619. doi: 10.1093/pcp/pcu191

Seki, M., Ohara, T., Hearn, T. J., Frank, A., da Silva, V. C. H., Caldana, C., et al. (2017) Adjustment of the Arabidopsis circadian oscillator by sugar signaling dictates the regulation of starch metabolism. *Sci. Rep.* 7, 8305. doi: 10.1038/s41598-017-08325-y

Thompson, M. V. (2006) Phloem: the long and the short of it. *Trends Plant Sci.* 11, 26–32. doi: 10.1016/j.tplants.2005.11.009
